# Estimating health state utility from activities of daily living in the French National Hospital Discharge Database: a feasibility study with head and neck cancer

**DOI:** 10.1186/s12955-019-1195-9

**Published:** 2019-07-25

**Authors:** Michaël Schwarzinger, Stéphane Luchini, for the EPICORL Study Group, Sylvain Baillot, Mélina Bec, Lynda Benmahammed, Caroline Even, Lionnel Geoffrois, Florence Huguet, Béatrice Le Vu, Laurie Lévy-Bachelot, Stéphane Luchini, Yoann Pointreau, Camille Robert, Luis Sagaon Teyssier, Antoine Schernberg, Michaël Schwarzinger, Stéphane Temam

**Affiliations:** 1 Translational Health Economics Network (THEN), 39 quai de Valmy, 75010 Paris, France; 2 Infection Antimicrobials Modeling & Evolution (IAME), UMR 1137, Institut National de la Santé et de la Recherche Médicale (INSERM), Université Paris Diderot, Sorbonne Paris Cité, Paris, France; 3 Aix-Marseille University (Aix-Marseille School of Economics), Centre National de la Recherche Scientifique and EHESS Marseille, Marseille, France

**Keywords:** Head and neck cancer, Health state utility, EQ-5D-3L, QALYs, Cost-effectiveness analysis, Activities of daily living, Item response theory, National hospital discharge database

## Abstract

**Background:**

Health state utility (HSU) is a core component of QALYs and cost-effectiveness analysis, although HSU is rarely estimated among a representative sample of patients. We explored the feasibility of assessing HSU in head and neck cancer from the French National Hospital Discharge database.

**Methods:**

An exhaustive sample of 53,258 incident adult patients with a first diagnosis of head and neck cancer was identified in 2010–2012. We used a cross-sectional approach to define five health states over two periods: three "cancer stages at initial treatment" (early, locally advanced or metastatic stage); a "relapse state" and otherwise a "relapse-free state" in the follow-up of patients initially treated at early or locally advanced stage. In patients admitted in post-acute care, a two-parameter graded response model (Item Response Theory) was estimated from all 144,012 records of six Activities of Daily Living (ADLs) and the latent health state scale underlying ADLs was calibrated with the French EQ-5D-3 L social value set. Following linear interpolation between all assessments of the patient, daily estimates of utility in post-acute care were averaged by health state, patient and month of follow-up. Finally, HSU was estimated by health state and month of follow-up for the whole patient population after controlling for survivorship and selection in post-acute care.

**Results:**

Head and neck cancer was generally associated with poor HSU estimates in a real-life setting. As compared to “distant metastasis at initial treatment”, mean HSU was higher in other health states, although numerical differences were small (0.45 versus around 0.54). It was primarily explained by the negative effects on HSU of an older age (38.4% aged ≥70 years in “early stage at initial treatment”) and comorbidities (> 50% in other health states). HSU estimates significantly improved over time in the “relapse-free state” (from 8 to 12 months of follow-up).

**Conclusions:**

HSU estimates in head and neck cancer were primarily driven by age at diagnosis, comorbidities, and time to assessment of cancer survivors. This feasibility study highlights the potential of estimating HSU within and across severe conditions in a systematic way at the national level.

**Electronic supplementary material:**

The online version of this article (10.1186/s12955-019-1195-9) contains supplementary material, which is available to authorized users.

## Background

Cost-effectiveness analysis is used in most high-income countries for pricing and reimbursement of new health interventions [1]. In such analysis, effectiveness is generally measured by Quality-Adjusted Life Years (QALYs) where the expected number of years to be lived in different health states is weighted by community preferences for each health state [1, 2]. However, these health state utility (HSU) estimates are typically among the most important but also uncertain drivers of cost-effectiveness results – a paradoxical situation that seems detrimental to fair pricing and reimbursement decisions across competing new health interventions.

There are multiple sources of variability in HSU estimates, although a general adherence to the same guidelines would purposely limit variability to patient surveys [1–3]. Indeed, if the same preference-based, generic health-related quality-of-life (HRQoL) instrument was administered in all patient surveys, then all HRQoL profiles of the patients could be similarly converted into HSU estimates with use of country-specific social value sets [4]. However, the variability of HSU estimates may still remain considerable due to the scarcity, small sample size, and lack of representativeness of patient surveys as recently illustrated in the context of relapsed/metastatic head and neck cancer [5–7].

In a systematic review of HSU estimates in head and neck cancer [8], Meregaglia and Cairns identified that only 12 patient surveys collected preference-based, generic HRQoL data. Most (9/12) patient surveys relied on the same EQ-5D-3L instrument [9], but none provided HSU estimates by cancer stage due to small sample sizes [8]. Otherwise, EQ-5D-3L data are increasingly collected along clinical trials [3]. However, HSU estimates lack representativeness due to the exclusion criteria applied to the patient population such as an older age or the presence of comorbidities [10–12]. Altogether, none of the patient surveys were conducted in France [8] and few French patients were recruited in international clinical trials (e.g., less than 20 patients in [12]). By default, a cost-effectiveness analysis conducted in the French healthcare context should further assume that patient surveys from other countries are representative of French patients [13].

In this study, we explored another route than patient surveys to estimate consistent HSU at the country level. More specifically, the French National Hospital Discharge database allows identifying all patients cared with a severe condition such as cancer as well as health states typically used in a cost-effectiveness analysis such as cancer stage at initial treatment and relapse in the follow-up. In addition, six Activities of Daily Living (ADLs) are systematically collected in patients admitted in post-acute care. Taking head and neck cancer as a case study, we developed a multi-step process to estimate HSU. Steps I and II consist of patient data organization of the French National Hospital Discharge database including selection of incident patients and definition of five core health states over two periods (initial treatment and follow-up). Step III enables utility to be estimated daily from all records of ADLs in post-acute care with use of Item Response Theory [14]. Step IV enables HSU to be estimated by patient and month of follow-up in the whole patient population after controlling for survivorship and selection in post-acute care.

## Methods

### Data source

The data source was the French National Hospital Discharge (PMSI) database in the years 2008 to 2013. The database contains all public and private hospital claims for acute and post-acute care. The standardized discharge summary includes: patient’s demographics (gender, age, postal code of residency); primary and associated discharge diagnosis codes according to the WHO International Classification of Diseases, tenth revision (ICD-10); medical procedures performed; length of stay; and discharge mode (including in-hospital death). In addition, six ADLs are systematically scored at admission in post-acute care and then every week until hospital discharge (Table 1). For research purposes, all hospital discharge data of the patient could be traced in 2008–2013 with use of an unique anonymous identifier [15, 16].

**Table 1:** Activities of Daily Living (ADL) recorded in post-acute care among head and neck cancer patients (*n* = 144,012)

ADL	Actions assessed		Severity level^a^	n (%)
1. Dressing or bathing	2	Dressing of the upper body	Independence	71,810 (49.86)
		Dressing of the lower body (including shoes)	Supervision	23,073 (16.02)
	2	Bathing of the upper body (including shaving or grooming)	Partial dependence	22,521 (15.64)
		Bathing of the lower body (including genital area)	Total dependence	26,608 (18.48)
2. Functional mobility (transferring)	5	Moving in and out of bed or chair	Independence	71,523 (49.66)
		Moving in and out of bathtub or shower	Supervision	26,499 (18.40)
		Getting on and off the toilet	Partial dependence	19,173 (13.31)
		Walking or using wheelchair	Total dependence	26,817 (18.62)
		If walking, climbing and descending staircase		
3. Self-feeding	3	Using kitchen utensils	Independence	43,057 (29.90)
		Chewing	Supervision	31,600 (21.94)
		Swallowing	Partial dependence	26,139 (18.15)
			Total dependence	43,216 (30.01)
4. Continence	2	Exercising complete self-control over urination	Independence	90,564 (62.89)
			Supervision	20,039 (13.91)
		Exercising complete self-control over defecation	Partial dependence	12,472 (8.66)
			Total dependence	20,937 (14.54)
5. Social interaction	1	Social functioning	Independence	73,436 (50.99)
			Supervision	42,415 (29.45)
			Partial dependence	18,109 (12.57)
			Total dependence	10,052 (6.98)
6. Communication	2	Understanding audio or visual communication	Independence	70,230 (48.77)
			Supervision	41,073 (28.52)
		Using verbal or nonverbal communication	Partial dependence	20,314 (14.11)
			Total dependence	12,395 (8.61)

^a^Independence: does not need from a third party; Supervision: needs help from a third party without physical intervention; Partial dependence: third party necessary to complete at least one action; Total dependence: third party necessary to complete at least one action

### Step I: selection of incident patients

We included all adults residing in metropolitan France and discharged with a primary or associated discharge diagnosis code of head and neck squamous-cell carcinoma (ICD-10: C00-C06; C09-C14; C30.0; C31; C32) in the years 2008 to 2012. We selected incident cases in 2010–2012 after excluding all prevalent cases in 2008–2009 [17, 18]. In addition, we excluded all incident cases recorded with a personal history of cancer to minimize a possible misclassification of a relapse. The coding dictionary of all variables used in this study is provided in Additional file 1**:** Table S1.

### Step II: health state definition over two periods

Most patients with head and neck cancer are diagnosed at locally advanced stage [19] and receive combined-modality treatments over a few months to decrease the high risk of relapse in the short-term [20]. In patient surveys, EQ-5D-3L was mostly (8/9) assessed after initial treatment in relapse-free patients [8]. In accordance with the usual design of patient surveys, ADLs are recorded in post-acute care in the French National Hospital Discharge database, although we aimed at expanding utility assessment to several health states including a relapse state [5–8].

We used a cross-sectional approach to define five health states over two periods: three cancer stages at initial treatment (early, locally advanced or metastatic stage) [20]; a relapse state and otherwise a relapse-free state in the follow-up. The initial treatment phase was defined by the first 6 months after diagnosis to encompass various lengths of combined-modality treatments [21] and related post-acute care. Cancer stage was identified at initial treatment from medical information that is consistently recorded at hospital [22]: a metastatic stage was identified by any record of distant metastasis; in absence of distant metastasis, a locally advanced stage was identified by any diagnosis indicating locoregional extension (e.g., lymph nodes) or any initial treatment eliminating an early stage (e.g., chemotherapy) [20]; and an early stage was considered by default in other patients.

Patients identified at the metastatic stage at initial treatment had poor prognosis and were followed in the same health state until end of follow-up. Other patients identified at early or locally advanced stage became at risk of relapse after 6 months. Relapse was identified by the first record of a local relapse (i.e., primary discharge diagnosis identical to the original cancer site) or a new event indicative of extension (i.e., distant metastasis, locoregional extension, or chemotherapy). Relapsing patients had poor prognosis and were followed in the same health state until end of follow-up. Other patients were considered relapse-free in the follow-up, starting from 6 months after diagnosis to end of follow-up.

Overall mortality was assessed from in-hospital death records as well as deaths outside hospital with right-censoring for all patients at July 1, 2013 (Additional file 1: Methods). The Kaplan-Meier method was used to test the association of health state with survival over a maximum follow-up of 12 months. The Fine and Gray method was used to test the association of health state with post-acute care admission, where deaths without post-acute care were considered as competing events [23].

### Step III: utility estimation over time in post-acute care

Six ADLs are systematically scored at admission in post-acute care and then every week until hospital discharge: 4 self-care tasks (dressing/bathing; functional mobility; self-feeding; continence); social interaction; and communication (Table 1). Each ADL is scored on the same 4-level scale (0 = total dependence, 1 = partial dependence, 2 = supervision, or 3 = independence).

All records of ADLs in post-acute care were analyzed with Item Response Theory [14]. We estimated a two-parameter graded response model [24], in which ordinal scores on ADLs are assumed to be a logistic function of a latent health state scale (i.e., the probability of a higher score on each ADL increases as the latent health state increases). The model is specified as follows:

$$ {P}_{ijk\left({X}_j\ge k|{\theta}_i,{\alpha}_j\right)}=\frac{e^{\alpha_j\left({\theta}_i-{\beta}_{jk}\right)}}{1+{e}^{\alpha_j\left({\theta}_i-{\beta}_{jk}\right)}} $$

where *P*_*ijk*_ is the cumulative probability that patient *i* receives a score of *k* or above (k = 0, 1, 2, 3) on ADL *j* (j = 1, 2, 3, 4, 5, 6); *θ*_*i*_ represents the latent health state value of patient *i*; *α*_*j*_ is the slope parameter of ADL *j* and indicates the ability of ADL *j* to discriminate patients on the latent health state scale; and *β*_*jk*_ is the threshold parameter of ADL *j* for score *k* or above relative to lower scores and indicates the value at which a patient has a 50% chance of scoring *k* or above on the latent health state scale (i.e., k-1 threshold parameters are estimated).

We assessed the unidimensionality of the latent health state scale, i.e., the assumption that all ADLs measure a single construct of health state, by examining the eigenvalues of the polychoric correlation matrix [14]. Assuming a perfect correlation between the latent health state scale and the French EQ-5D-3L social value set, we computed an ADL-related utility scale calibrated on the worst (− 0.53) and best (1.00) anchors of the French EQ-5D-3L social value set [25]:

$$ {\hat{U}}_{EQ-5D}^{IRT}=\left[\frac{\left({\hat{U}}_{RAW}^{IRT}-\min {\hat{U}}_{RAW}^{IRT}\right)}{\left(\max {\hat{U}}_{RAW}^{IRT}-\min {\hat{U}}_{RAW}^{IRT}\right)}\times \left(1+0.53\right)-0.53\right] $$

Finally, patients may have repeated assessments (i.e., weekly assessments during the same hospital stay and/or multiple hospital stays in post-acute care) and ADL-related utility was linearly interpolated on a daily basis between all assessments from first to last record of the patient in post-acute care.

### Step IV: HSU estimation by month of follow-up in the whole patient population

We controlled for a possible survivorship effect on utility by estimating HSU by patient and month of follow-up in each health state. We expanded on previous cross-sectional approach (Step II) to define 48 subpopulations consisting of all patients alive at the beginning of each month of follow-up in a given health state (from 1 to 6 months in early or locally advanced stage at initial treatment; and from 1 to 12 months in the three other health states). In each subpopulation, we identified all patients recorded in post-acute care and HSU was computed by the average of daily ADL-related utility estimates in the month per patient. In the best case scenario with complete daily estimates (*n* = 30 in the month), HSU represented the area-under-the-curve utility estimate of the patient. In the worst case scenario with a single daily estimate (*n* = 1 in the month), we assumed that ADL-related utility of the patient was uniform over the month.

Then, we estimated HSU for the whole subpopulation with use of a two-step selection model [26]. In the first step, the selection equation is a binary probit regression estimating the probability of a patient to be recorded in post-acute care in the month:

$$ P\left(\mathrm{post}-\mathrm{acute}\ \mathrm{care}=1\right)=\Phi \left(\beta {\mathrm{X}}_i\right) $$

where *i* represents patients, X represents a vector of covariates, and ϕ is the cumulative distribution function of the normal distribution. Since our general aim was to improve inference rather than efficiency [27], we used a large set of covariates including time-independent covariates (demographics; tobacco smoking, alcohol use; year at diagnosis, primary head and neck cancer site, second synchronous head and neck cancer) and time-dependent covariates recorded before or during the given month (admission to a public teaching hospital, comprehensive cancer care center, private clinic; second primary cancer other than head and neck cancer [28, 29], each comorbidity of the Charlson comorbidity index other than cancer [30, 31], depression; palliative care) (Additional file 1: Table S1).

In the second step, the outcome equation is a standard OLS regression estimating HSU in post-acute care while controlling for selection bias:

$$ {\mathrm{HSU}}_i={\gamma \mathrm{Y}}_i+\lambda {\mathrm{IMR}}_i $$

where *i* represents patients, Y represents a vector of covariates, and IMR (for inverse Mills ratio) is the correction factor of selection bias calculated from the probit model at *β*X_*i*_ in the selection equation. Selection bias was assessed by testing the null that the coefficient of IMR *λ* = 0. We used the set of covariates of the selection equation, although some covariates that were assumingly less related to HSU were removed from the outcome equation (region of residency, risk factors, previous admission to several types of hospital) [32]. Since the set of covariates of the selection equation was defined in all patients, we used the outcome equation to impute HSU in all patients unrecorded in post-acute care in the month.

All statistical analyses were performed with SAS 9.4 including PROC IRT for estimating the two-parameter graded response model.

## Results

### Step I: selection of incident patients

Of the 27.3 million adults discharged from all French hospitals in 2008–2012, 134,324 (0.49%) had a diagnosis of head and neck cancer (Additional file 1: Table S2). Of them, 53,258 (40.4%) were considered incident cases in 2010–2012.

### Step II: health state definition over two periods

**Fig. 1:**
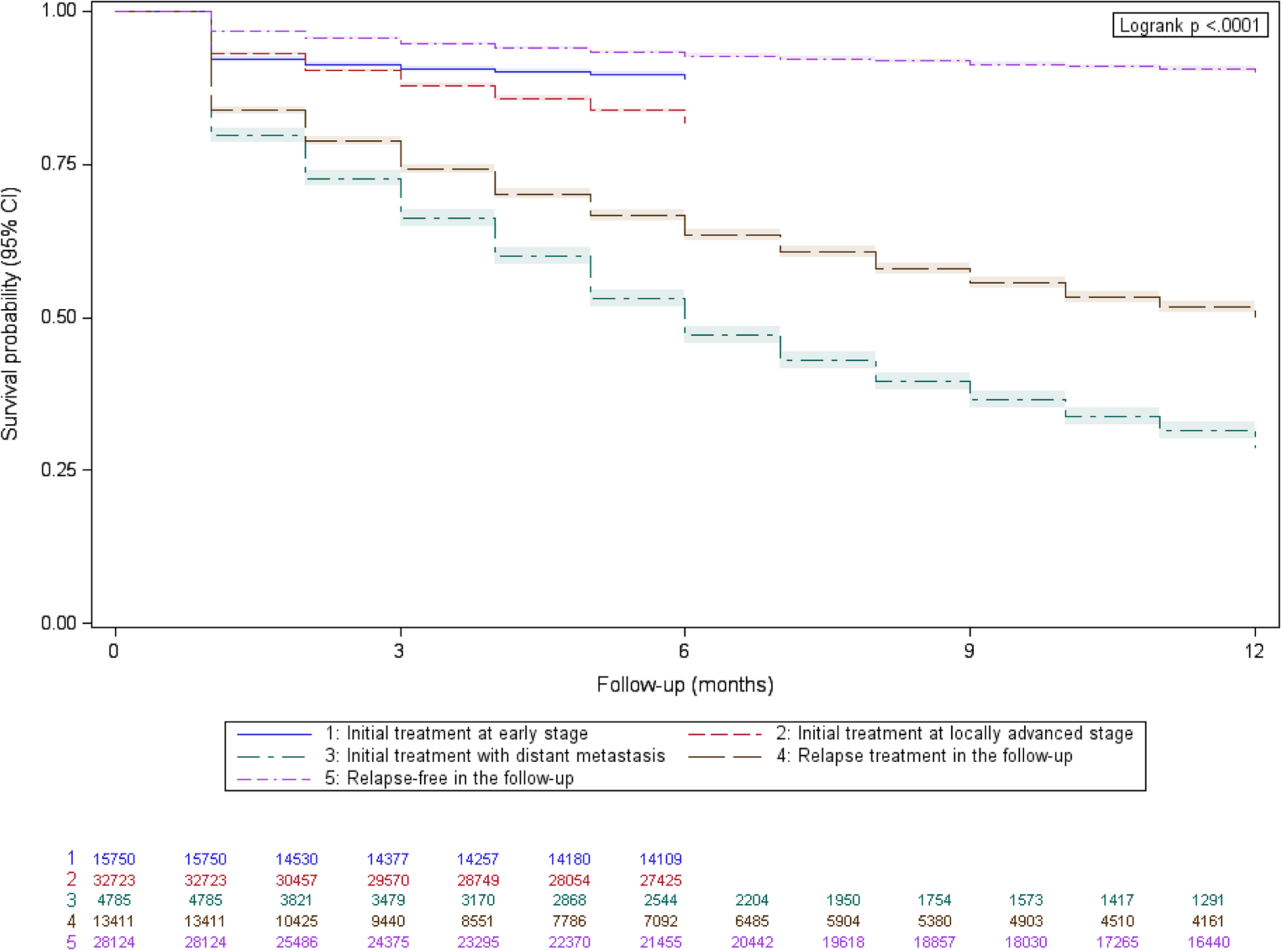
Survival according to health state in head and neck cancer

Five health states were defined over two periods: initial treatment and follow-up. Health states were significantly associated with survival (Fig. 1). Patients with distant metastasis at initial treatment or relapsing in the follow-up had the worst prognosis. Patients initially treated at early stage had better prognosis as compared to patients treated at locally advanced stage. Patients in a relapse-free state had the best prognosis.

**Fig. 2:**
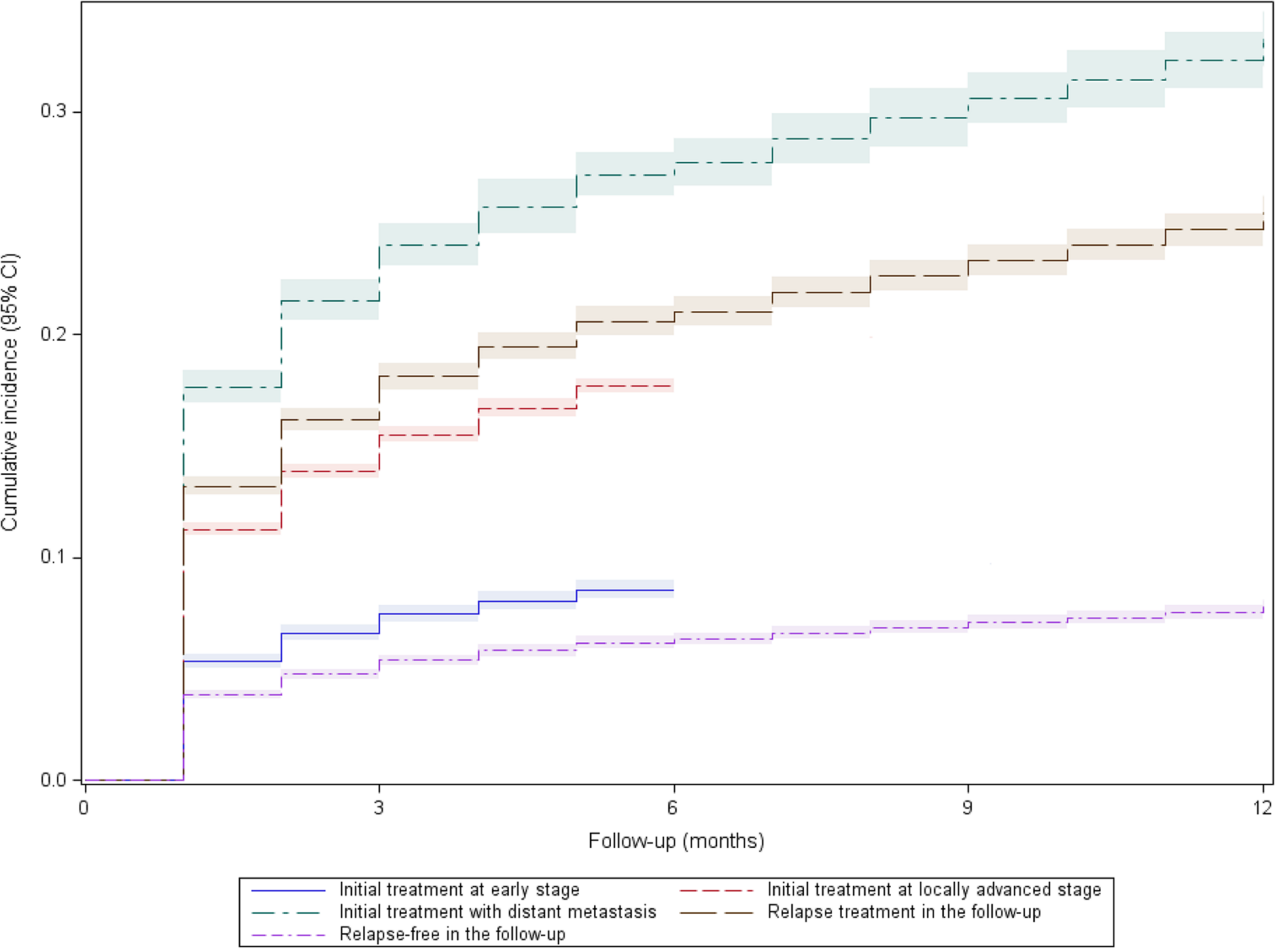
Admission in post-acute care according to health state in head and neck cancer

Health states of poor prognosis were significantly associated with higher admission rates in post-acute care (Fig. 2). At initial treatment, patients with distant metastasis were 3.5 times more likely to be admitted in post-acute care as compared to patients at early stage (HR = 3.54, 95% CI 3.31–3.80). In the follow-up, relapsing patients were 3.6 times more likely to be admitted in post-acute care as compared to patients in a relapse-free state (HR = 3.62, 95% CI 3.42–3.82).

### Step III: utility estimation over time in post-acute care

**Fig. 3:**
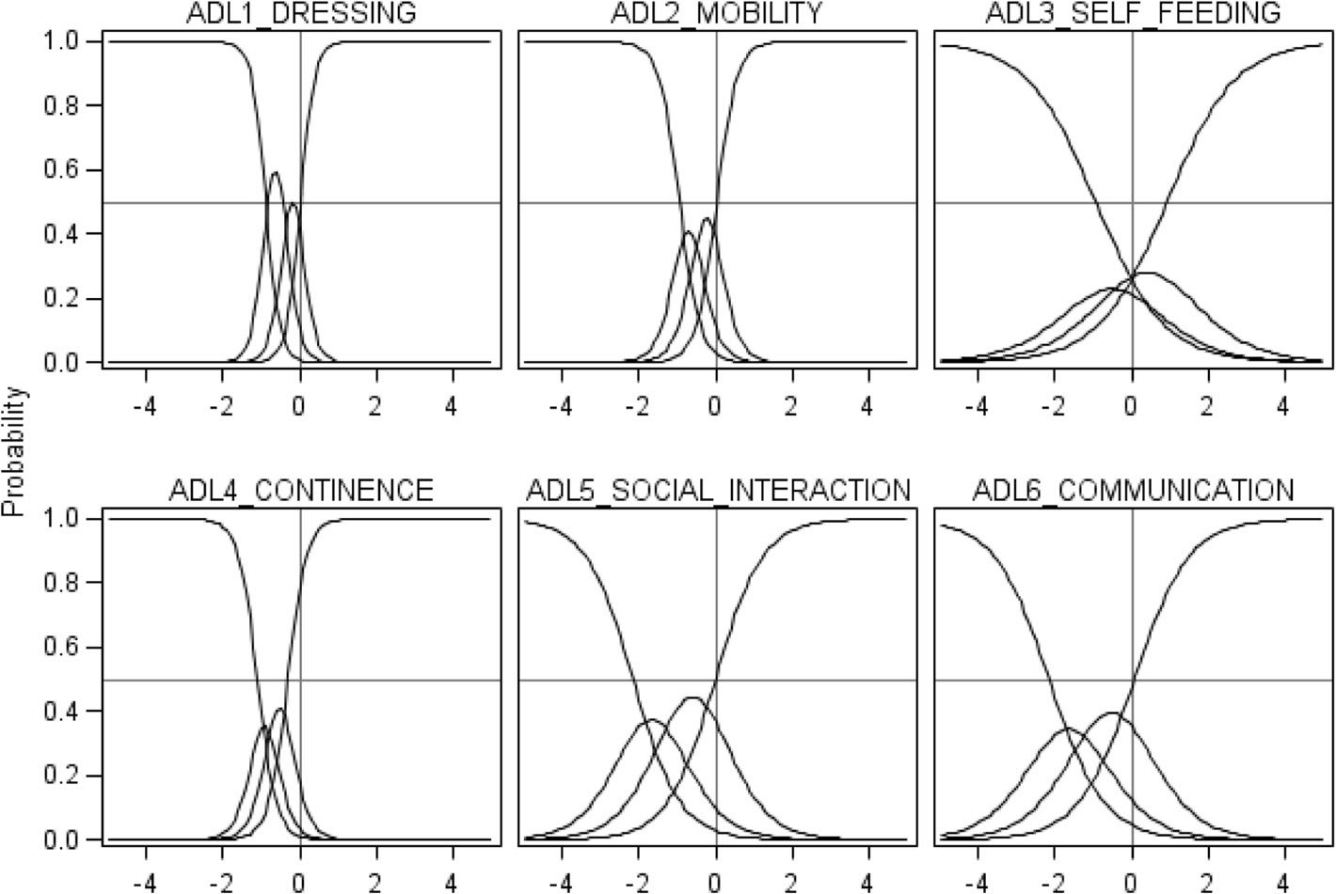
Characteristic curves of 6 Activities of Daily Living (ADL) recorded in post-acute care (*n* = 144,006). The trait on the horizontal axis is an arbitrarily scaled representation of the latent health state scale. As the value of the latent health state increases, the probability of a higher score on each ADL increases. The relative concentration of the curves reflects the relatively high discriminative ability of an ADL. On the contrary, the relative spread of the curves reflects the relatively low discriminative ability of an ADL

Six ADLs were assessed at 144,012 points in time in post-acute care (Table 1). The two-parameter graded response model fitted very well all records of ADLs. The unidimensionality of the latent health scale was supported by the examination of eigenvalues: the first eigenvalue (4.0) explained 66.8% of the variance; the second eigenvalue was below 1.0; and the ratio of the first and second eigenvalues (4.6) was above 3 (Additional file 1: Table S3). In addition, all slope parameters were above 1 indicating that all ADLs were informative regarding the latent health state scale (Additional file 1: Table S4). The assessment of dressing/bathing was the most informative on the latent health state (slope = 5.50; range between threshold parameters = 4.95) (Fig. 3). The assessment of self-feeding was the least informative on the latent health state (slope = 1.12; range between threshold parameters = 2.09). Most (14/18) threshold parameters were below 0 indicating that ADLs were generally more informative on poor health states.

Following calibration of the latent health state scale on the French EQ-5D-3L social value set, the ADL-related utility had a mean (std) of 0.44 (0.40) and a median (IQR) of 0.47 (0.18–0.76). ADL-related utility estimates were completed on a daily basis with use of linear interpolation between all assessments of the patient in post-acute care. The final dataset included 1,032,301 daily estimates of ADL-related utility in post-acute care with a mean (std) of 0.44 (0.38) and a median (IQR) of 0.47 (0.18–0.74).

### Step IV: HSU estimation by month of follow-up in the whole patient population

**Fig. 4:**
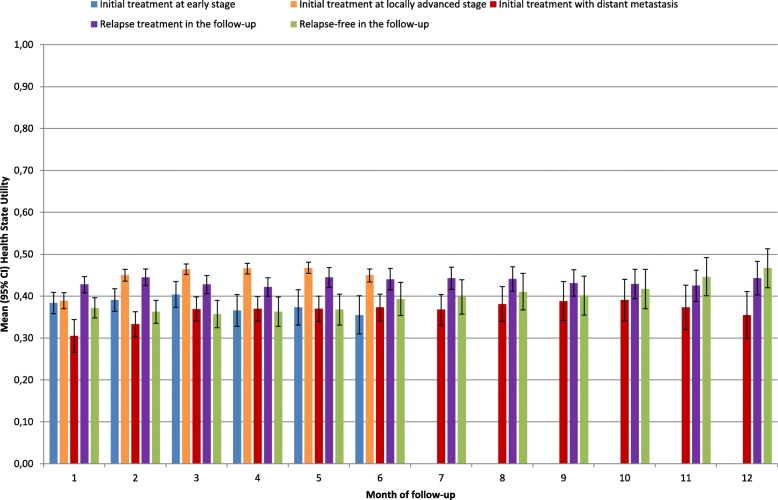
Health state utility, by health state and month of follow-up of head and neck cancer patients in post-acute care

Daily estimates of ADL-related utility in post-acute care were averaged into HSU estimates by health state, patient and month of follow-up. Patients initially treated at early stage had surprisingly lower HSU estimates than patients at locally advanced stage and a selection bias in post-acute care was suspected (Fig. 4).

**Fig. 5:**
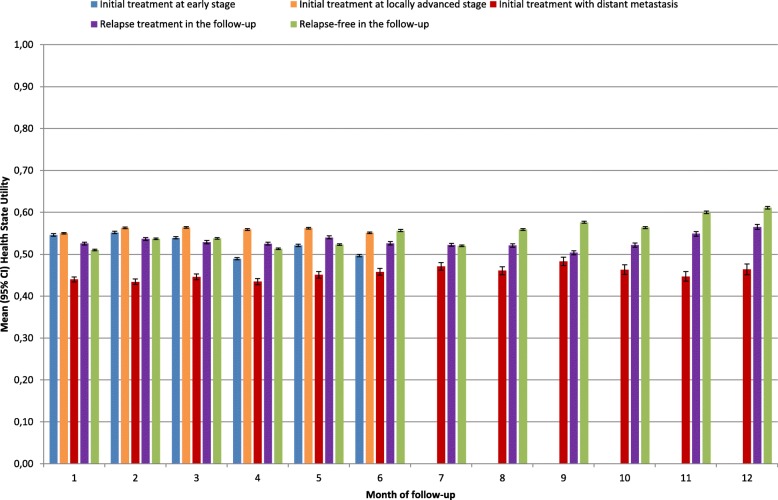
Health state utility, by health state and month of follow-up of all head and neck cancer patients

Considering all patients alive at the beginning of the month in a given health state, two-step selection models were carried out by health state and month of follow-up (parameter estimates are provided at first and last month of follow-up for the 5 health states in Additional file 1: Tables S5–S14). Overall, HSU estimates significantly increased for each health state and month of follow-up after controlling for selection in post-acute care (Fig. 5). A selection bias was primarily found in patients initially treated at early stage (*p* < 0.05 for 4 out of 6 months of follow-up) or locally advanced stage (*p* < 0.05 for 6 out of 6 months of follow-up) (Additional file 1: Table S15), although HSU estimates remained lower in patients initially treated at early stage as compared to locally advanced stage. Patients initially treated with distant metastasis had the worst HSU estimates at all months of follow-up. Patients in a relapse-free state had the best HSU estimates after 8 months of follow-up, with an increasing trend from 8 to 12 months of follow-up (max HSU of 0.61 at 12 months of follow-up).

HSU summary statistics were computed over the all period of follow-up (Table 2). As compared to the health state “distant metastasis at initial treatment” (mean HSU = 0.45), other health states were associated with a better mean HSU, although numerical differences were small around 0.54. It was primarily explained by the negative effects on HSU of an older age in the health state “early stage at initial treatment” (38.4% patients were aged ≥70 years) and comorbidities (> 50%) in other health states.

**Table 2:** Summary statistics of health state utility (HSU) in head and neck cancer

		Initial treatment at early stage		Initial treatment at locally advanced stage		Initial treatment with distant metastasis		Relapse treatment in the follow-up		Relapse-free in the follow-up	
		n (%)	Mean (std)	n (%)	Mean (std)	n (%)	Mean (std)	n (%)	Mean (std)	n (%)	Mean (std)
Overall		87,965 (100)	0.525 (0.183)	181,094 (100)	0.558 (0.174)	33,573 (100)	0.451 (0.224)	91,929 (100)	0.530 (0.186)	256,816 (100)	0.546 (0.170)
Age at diagnosis	18–49	10,519 (12.0)	0.699 (0.101)	25,615 (14.1)	0.647 (0.114)	3,917 (11.7)	0.555 (0.187)	14,316 (15.6)	0.590 (0.160)	36,532 (14.2)	0.635 (0.117)
	50–54	9,271 (10.5)	0.700 (0.101)	29,629 (16.4)	0.632 (0.122)	5,298 (15.8)	0.524 (0.193)	16,529 (18.0)	0.582 (0.162)	36,010 (14.0)	0.646 (0.131)
	55–59	11,770 (13.4)	0.624 (0.108)	36,029 (19.9)	0.617 (0.130)	6,808 (20.3)	0.439 (0.198)	19,071 (20.7)	0.569 (0.164)	43,821 (17.1)	0.638 (0.128)
	60–64	13,182 (15.0)	0.554 (0.113)	32,872 (18.2)	0.562 (0.145)	6,578 (19.6)	0.486 (0.213)	16,389 (17.8)	0.498 (0.181)	43,704 (17.0)	0.540 (0.121)
	65–69	9,443 (10.7)	0.533 (0.122)	20,237 (11.2)	0.519 (0.148)	4,290 (12.8)	0.427 (0.220)	9,753 (10.6)	0.510 (0.178)	28,573 (11.1)	0.531 (0.131)
	70–74	9,262 (10.5)	0.415 (0.140)	14,551 (8.0)	0.450 (0.167)	2,627 (7.8)	0.392 (0.217)	7,077 (7.7)	0.467 (0.197)	23,060 (9.0)	0.446 (0.137)
	75–79	9,864 (11.2)	0.422 (0.140)	11,333 (6.3)	0.416 (0.182)	1,994 (5.9)	0.286 (0.241)	4,852 (5.3)	0.432 (0.198)	21,273 (8.3)	0.428 (0.163)
	80+	14,654 (16.7)	0.316 (0.151)	10,828 (6.0)	0.306 (0.231)	2,061 (6.1)	0.269 (0.248)	3,942 (4.3)	0.317 (0.198)	23,843 (9.3)	0.324 (0.171)
Comorbidities	0	46,845 (53.3)	0.571 (0.145)	80,616 (44.5)	0.602 (0.128)	9,555 (28.5)	0.501 (0.176)	28,200 (30.7)	0.568 (0.145)	127,033 (49.5)	0.583 (0.125)
	1	21,650 (24.6)	0.519 (0.171)	53,581 (29.6)	0.567 (0.158)	11,009 (32.8)	0.478 (0.202)	29,366 (31.9)	0.545 (0.171)	68,498 (26.7)	0.547 (0.157)
	2	10,886 (12.4)	0.459 (0.206)	26,311 (14.5)	0.515 (0.193)	6,914 (20.6)	0.428 (0.234)	17,726 (19.3)	0.512 (0.197)	33,774 (13.2)	0.505 (0.198)
	≥3	8,584 (9.8)	0.369 (0.243)	20,586 (11.4)	0.417 (0.244)	6,095 (18.2)	0.349 (0.275)	16,637 (18.1)	0.456 (0.232)	27,511 (10.7)	0.429 (0.255)

## Discussion

Although many Health Technology Assessment bodies (such as the French HAS [13]) have deemed QALYs the principal measure of effectiveness, still only a limited number of studies report QALYs based on actual assessments of preference-based, generic HRQoL among a representative sample of patients. The assessment of new immunotherapy for relapsed/metastatic head and neck cancer provides a pressing example [5–7] since few patient surveys were conducted and none provided HSU estimates by cancer stage due to small sample sizes [8].

In this study, we explored another route than patient surveys to estimate consistent HSU at the country level. On the one hand, all incident patients diagnosed with head and neck cancer in France were identified from the French National Hospital Discharge database. Five health states could be reliably defined over time for the whole patient population and we found expectedly that relapsed/metastatic patients had poor prognosis. On the other hand, ADLs rather than the recommended EQ-5D-3L instrument are recorded and we had to develop a multi-step process to transform ADLs records in post-acute care into consistent HSU estimates representative of the whole patient population.

One of the main study results is that head and neck cancer was generally associated with poor HSU estimates in a real-life setting since mean HSU ranged from 0.45 for “distant metastasis at initial treatment” to around 0.54 for other health states (early or locally advanced stage at initial treatment; relapse state and otherwise relapse-free state in the follow-up) with “minimally important differences” (< 0.06) between health states [33]. In comparison, EQ-5D-3L utility estimates were much higher in most (8/9) surveys conducted in relapse-free patients (median (IQR) sample size of 79 (28–112) patients), with a median (IQR) utility of 0.80 (0.78–0.84) for patients aged 63 years on average [8]. EQ-5D-3L utility estimates were also higher in one longitudinal study of 81 patients diagnosed at early/locally advanced stage and aged ≥65 years (median (IQR) utility of 0.66 (0.55–0.76) at diagnosis and 0.64 (0.00–0.74) at 12 months of follow-up) [34]. EQ-5D-3L utility estimates were also higher in patients selected in clinical trials, with a mean (std) utility of 0.79 (0.18) in 715 patients initially treated at locally advanced stage [11] and 0.68 (0.28) in 120 relapsed/metastatic patients [12]. While attention was drawn on the expected variability of EQ-5D-3L utility estimates with community preferences of the country [4, 8], our study results suggest that the lack of representativeness of patient surveys should be of primary concern since the usual recruitment of younger patients with less comorbidities may lead to overly optimistic HSU estimates.

Another main study result is that HSU estimates significantly improved over time in patients in a relapse-free state (from 8 to 12 months of follow-up) in agreement with HRQoL improvements found over longer periods of time in cancer survivors [35, 36]. In comparison, the time to assessment of EQ-5D-3L varied dramatically within and between surveys conducted in relapse-free patients (i.e., from months to years after diagnosis) [8]. On the one hand, a longer time to assessment in cross-sectional patient surveys may also explain our lower HSU estimate since follow-up was limited to 1 year and accounted for utility at each month of follow-up in the relapse-free state. On the other hand, our study results suggest that time to assessment should be better accounted for or even standardized to achieve comparable HSU estimates between patient surveys. Otherwise, we found that HSU estimates did not improve over time in health states other than the relapse-free state. Similarly, no significant changes in EQ-5D-3L utility were found over time in old patients diagnosed at early/locally advanced stage [34], trial patients initially treated at locally advanced stage [11], or trial patients treated at relapsed/metastatic stage [12]. Altogether, it suggests that EQ-5D-3L social value sets exhibit a poor responsiveness to change during treatment in head and neck cancer [37].

The strengths of this nationwide study outline its limitations. Indeed, this study is a secondary analysis of the French National Hospital Discharge database and therefore all measurements relied on administrative records with possible misclassification. Regarding health state definition, TNM cancer staging is not recorded in the standardized discharge summary and we constructed a composite variable to identify three cancer stages at initial treatment. Overall, 37,508 (70.4%) of 53,258 patients were identified at a late stage at initial treatment (Fig. 1), in agreement with previous reports of cancer registries [19]. However, we could no longer estimate HSU related to the treatment modalities since this information was already used to construct the composite variable of cancer stage.

Regarding utility estimation, ADL scores contribute with discharge diagnoses, rehabilitation procedures, and age to the hospital billing system in post-acute care. Accordingly, the completion rate of ADLs was extremely high (> 99%), although a recording bias towards more severe scores is possible and could lead to lower HSU estimates. In absence of mapping studies of ADLs into EQ-5D-3L social value sets [38–40], a latent health state scale was estimated from all records of ADLs with use of Item Response Theory and then calibrated on the worst (− 0.53) and best (1.00) anchors of the French EQ-5D-3L social value set [25]. Such approach was supported by the conceptual overlap between ADLs and the EQ-5D-3L instrument regarding dimensions and their ordinal scoring as well as the unidimensionality of the latent health state scale underlying ADLs. However, the calibration implies a perfect correlation of the latent health state scale with the French EQ-5D-3L social value set and the distribution of ADL-related utility should be cross-validated with a mapping study conducted in post-acute care. In the following steps, we made a full use of the repeated assessments of ADLs by patient (linear interpolation of ADL-related utility on a daily basis and then average by month of follow-up) that resulted in smoothed and generally unimodal distributions of utility in the 48 subpopulations. In particular, we found limited evidence of a ceiling effect in post-acute care (utility of 1.00 for 8.6% of 40,812 patients selected in all 48 subpopulations; at maximum, 13.8% of 290 relapse-free patients at 12 months of follow-up) [37].

## Conclusions

HSU estimates in head and neck cancer were primarily driven by age at diagnosis, comorbidities, and time to assessment of cancer survivors. This feasibility study highlights the potential of estimating HSU within and across severe conditions in a systematic way at the national level. While the multi-step process to estimate HSU was developed with use of the French National Hospital Discharge database, it may generalize to other Hospital Discharge databases including a systematic assessment of ADLs for billing purposes.

## Additional file

Additional file 1: Additional Methods. Imputation of mortality outside hospital. **Table S1.** Coding dictionary. **Table S2.** Study flowchart. **Table S3.** Eigenvalues of the Polychoric Correlation Matrix (two-parameter graded response model). **Table S4.** Parameter estimates (two-parameter graded response model). **Table S5.** Parameter estimates of the two-step selection model for “initial treatment at early stage” at 1 month of follow-up. **Table S6.** Parameter estimates of the two-step selection model for “initial treatment at early stage” at 6 months of follow-up. **Table S7.** Parameter estimates of the two-step selection model for “initial treatment at locally advanced stage” at 1 month of follow-up. **Table S8.** Parameter estimates of the two-step selection model for “initial treatment at locally advanced stage” at 6 months of follow-up. **Table S9**. Parameter estimates of the two-step selection model for “initial treatment with distant metastasis” at 1 month of follow-up. **Table S10.** Parameter estimates of the two-step selection model for “initial treatment with distant metastasis” at 12 months of follow-up. **Table S11.** Parameter estimates of the two-step selection model for “relapse treatment in the follow-up” at 1 month of follow-up. **Table S12.** Parameter estimates of the two-step selection model for “relapse treatment in the follow-up” at 12 months of follow-up. **Table S13.** Parameter estimates of the two-step selection model for “relapse-free in the follow-up” at 1 month of follow-up. **Table S14**. Parameter estimates of the two-step selection model for “relapse-free in the follow-up” at 12 months of follow-up. **Table S15**. Selection bias in post-acute care by health state and month of follow-up. (DOCX 208 kb)

## Data Availability

Data sharing of the French National Hospital Discharge database or any related dataset with de-identified data such as the dataset generated for the current study is forbidden by law.

## Abbreviations

ADLs: Activities of daily living on 6 dimensions
EQ-5D-3L: EuroQol 5 dimensions 3 levels instrument
HRQoL: Health-related quality of life
HSU: Health state utility
ICD-10: International Classification of Diseases, tenth revision
IQR: Interquartile range
IRT: Item Response Theory
OLS: Ordinary least squares *regression*
PMSI: Programme de médicalisation des systèmes d’information
QALYs: Quality-Adjusted Life Years

## References

[1] Barnieh L, Manns B, Harris A, Blom M, Donaldson C, Klarenbach S, Husereau D, Lorenzetti D, Clement F (2014). A synthesis of drug reimbursement decision-making processes in organisation for economic co-operation and development countries. Value Health.

[2] Sanders GD, Neumann PJ, Basu A, Brock DW, Feeny D, Krahn M, Kuntz KM, Meltzer DO, Owens DK, Prosser LA (2016). Recommendations for conduct, methodological practices, and reporting of cost-effectiveness analyses: second panel on cost-effectiveness in health and medicine. JAMA.

[3] Wolowacz SE, Briggs A, Belozeroff V, Clarke P, Doward L, Goeree R, Lloyd A, Norman R (2016). Estimating health-state utility for economic models in clinical studies: an ISPOR good research practices task force report. Value Health.

[4] Xie F, Gaebel K, Perampaladas K, Doble B, Pullenayegum E (2014). Comparing EQ-5D valuation studies: a systematic review and methodological reporting checklist. Med Decis Mak.

[5] Nivolumab for treating squamous cell carcinoma of the head and neck after platinumbased chemotherapy. https://www.nice.org.uk/guidance/TA490/chapter/1-Recommendations. Accessed 20 Jul 2019.

[6] Ward MC, Shah C, Adelstein DJ, Geiger JL, Miller JA, Koyfman SA, Singer ME (2017). Cost-effectiveness of nivolumab for recurrent or metastatic head and neck cancer. Oral Oncol.

[7] Tringale KR, Carroll KT, Zakeri K, Sacco AG, Barnachea L, Murphy JD (2018). Cost-effectiveness analysis of Nivolumab for treatment of platinum-resistant recurrent or metastatic squamous cell carcinoma of the head and neck. J Natl Cancer Inst.

[8] Meregaglia M, Cairns J (2017). A systematic literature review of health state utility values in head and neck cancer. Health Qual Life Outcomes.

[9] Brooks R (1996). EuroQol: the current state of play. Health Policy.

[10] Del Barco ME, Mesia R, Adansa Klain JC, Vazquez Fernandez S, Martinez-Galan J, Pastor Borgonon M, Gonzalez-Rivas C, Caballero Daroqui J, Berrocal A, Martinez-Trufero J (2016). Phase II study of panitumumab and paclitaxel as first-line treatment in recurrent or metastatic head and neck cancer. TTCC-2009-03/VECTITAX study. Oral Oncol.

[11] Truong MT, Zhang Q, Rosenthal DI, List M, Axelrod R, Sherman E, Weber R, Nguyen-Tan PF, El-Naggar A, Konski A (2017). Quality of life and performance status from a substudy conducted within a prospective phase 3 randomized trial of concurrent accelerated radiation plus cisplatin with or without Cetuximab for locally advanced head and neck carcinoma: NRG oncology radiation therapy oncology group 0522. Int J Radiat Oncol Biol Phys.

[12] Harrington KJ, Ferris RL, Blumenschein G, Colevas AD, Fayette J, Licitra L, Kasper S, Even C, Vokes EE, Worden F (2017). Nivolumab versus standard, single-agent therapy of investigator's choice in recurrent or metastatic squamous cell carcinoma of the head and neck (CheckMate 141): health-related quality-of-life results from a randomised, phase 3 trial. Lancet Oncol.

[13] Haute Autorité de santé (HAS) (2011). Guide méthodologique - Choix méthodologiques pour l’évaluation économique à la HAS [Methodological guidance for health technology assessment submitted to the French Health Authority].

[14] De Ayala RJ (2009). The theory and practice of item response theory.

[15] Agence Technique de l’Information sur l’Hospitalisation (2010). Le décès dans le PMSI-MCO : validation et précautions d’utilisation. [Death in acute care : validation and precaution instructions].

[16] Agence Technique de l’Information sur l’Hospitalisation (2014). Aide à l’utilisation des informations de chaînage [How to use de-identified patient information].

[17] Schulman KL, Berenson K, Tina Shih YC, Foley KA, Ganguli A, de Souza J, Yaghmour NA, Shteynshlyuger A (2013). A checklist for ascertaining study cohorts in oncology health services research using secondary data: report of the ISPOR oncology good outcomes research practices working group. Value Health.

[18] Bagley SC, Altman RB (2016). Computing disease incidence, prevalence and comorbidity from electronic medical records. J Biomed Inform.

[19] Gatta G, Botta L, Sanchez MJ, Anderson LA, Pierannunzio D, Licitra L, Group EW (2015). Prognoses and improvement for head and neck cancers diagnosed in Europe in early 2000s: the EUROCARE-5 population-based study. Eur J Cancer.

[20] Gregoire V, Lefebvre JL, Licitra L, Felip E, Group E-E-EGW (2010). Squamous cell carcinoma of the head and neck: EHNS-ESMO-ESTRO clinical practice guidelines for diagnosis, treatment and follow-up. Ann Oncol.

[21] VanderWalde NA, Meyer AM, Liu H, Tyree SD, Zullig LL, Carpenter WR, Shores CD, Weissler MC, Hayes DN, Fleming M, Chera BS (2013). Patterns of care in older patients with squamous cell carcinoma of the head and neck: a surveillance, epidemiology, and end results-medicare analysis. J Geriatr Oncol.

[22] Amin MB, Edge S, Greene F, Byrd DR, Brookland RK, Washington MK, Gershenwald JE, Compton CC, Hess KR, Sullivan DC (2017). AJCC Cancer Staging Manual.

[23] Fine JP, Gray RJ (1999). A proportional hazards model for the subdistribution of a competing risk. J Am Stat Assoc.

[24] Samejima F (1969). Estimation of latent ability using a response pattern of graded scores. Psychometrika Monogr Suppl.

[25] Chevalier J, de Pouvourville G (2013). Valuing EQ-5D using time trade-off in France. Eur J Health Econ.

[26] Heckman JJ (1978). Dummy endogenous variables in a simultaneous equation system. Econometrica.

[27] Davidson R, MacKinnon J (1993). Estimation and inference in econometrics.

[28] Jegu J, Binder-Foucard F, Borel C, Velten M (2013). Trends over three decades of the risk of second primary cancer among patients with head and neck cancer. Oral Oncol.

[29] Jegu J, Colonna M, Daubisse-Marliac L, Tretarre B, Ganry O, Guizard AV, Bara S, Troussard X, Bouvier V, Woronoff AS, Velten M (2014). The effect of patient characteristics on second primary cancer risk in France. BMC Cancer.

[30] Charlson ME, Pompei P, Ales KL, MacKenzie CR (1987). A new method of classifying prognostic comorbidity in longitudinal studies: development and validation. J Chronic Dis.

[31] Boje CR (2014). Impact of comorbidity on treatment outcome in head and neck squamous cell carcinoma - a systematic review. Radiother Oncol.

[32] Sartori AE (2003). An estimator for some binary-outcome selection models without exclusion restrictions. Polit Anal.

[33] Pickard AS, Neary MP, Cella D (2007). Estimation of minimally important differences in EQ-5D utility and VAS scores in cancer. Health Qual Life Outcomes.

[34] Pottel L, Lycke M, Boterberg T, Pottel H, Goethals L, Duprez F, Rottey S, Lievens Y, Van Den Noortgate N, Geldhof K (2015). G-8 indicates overall and quality-adjusted survival in older head and neck cancer patients treated with curative radiochemotherapy. BMC Cancer.

[35] Weaver K. E., Forsythe L. P., Reeve B. B., Alfano C. M., Rodriguez J. L., Sabatino S. A., Hawkins N. A., Rowland J. H. (2012). Mental and Physical Health-Related Quality of Life among U.S. Cancer Survivors: Population Estimates from the 2010 National Health Interview Survey. Cancer Epidemiology Biomarkers & Prevention.

[36] Kim K, Kim JS (2017). Factors influencing health-related quality of life among Korean cancer survivors. Psychooncology.

[37] Schwenkglenks M, Matter-Walstra K (2016). Is the EQ-5D suitable for use in oncology? An overview of the literature and recent developments. Expert Rev Pharmacoecon Outcomes Res.

[38] Brazier JE, Yang Y, Tsuchiya A, Rowen DL (2010). A review of studies mapping (or cross walking) non-preference based measures of health to generic preference-based measures. Eur J Health Econ.

[39] Longworth L, Rowen D (2013). Mapping to obtain EQ-5D utility values for use in NICE health technology assessments. Value Health.

[40] Dakin H, Abel L, Burns R, Yang Y (2018). Review and critical appraisal of studies mapping from quality of life or clinical measures to EQ-5D: an online database and application of the MAPS statement. Health Qual Life Outcomes.

