# Supplementary material for: Estimating health state utility from activities of daily living in the French National Hospital Discharge Database: a feasibility study with head and neck cancer
Source: Health Qual Life Outcomes. 2019 Jul 25;17:129. doi: 10.1186/s12955-019-1195-9 (PMC6659251; doi:10.1186/s12955-019-1195-9)
Supplement: Supplementary file 1 — Additional Methods. Imputation of mortality outside hospital. Table S1. Coding dictionary. Table S2. Study flowchart. Table S3. Eigenvalues of the Polychoric Correlation Matrix (two-parameter graded response model). Table S4. Parameter estimates (two-parameter graded response model). Table S5. Parameter estimates of the two-step selection model for “initial treatment at early stage” at 1 month of follow-up. Table S6. Parameter estimates of the two-step selection model for “initial treatment at early stage” at 6 months of follow-up. Table S7. Parameter estimates of the two-step selection model for “initial treatment at locally advanced stage” at 1 month of follow-up. Table S8. Parameter estimates of the two-step selection model for “initial treatment at locally advanced stage” at 6 months of follow-up. Table S9. Parameter estimates of the two-step selection model for “initial treatment with distant metastasis” at 1 month of follow-up. Table S10. Parameter estimates of the two-step selection model for “initial treatment with distant metastasis” at 12 months of follow-up. Table S11. Parameter estimates of the two-step selection model for “relapse treatment in the follow-up” at 1 month of follow-up. Table S12. Parameter estimates of the two-step selection model for “relapse treatment in the follow-up” at 12 months of follow-up. Table S13. Parameter estimates of the two-step selection model for “relapse-free in the follow-up” at 1 month of follow-up. Table S14. Parameter estimates of the two-step selection model for “relapse-free in the follow-up” at 12 months of follow-up. Table S15. Selection bias in post-acute care by health state and month of follow-up. (DOCX 208 kb) [file 12955_2019_1195_MOESM1_ESM.docx]

**Estimating Health State Utility from Activities of Daily Living in the French National Hospital Discharge Database: a Feasibility Study with Head and Neck Cancer.** Schwarzinger M et al.

**Supplementary material contents**

[Additional Methods. Imputation of mortality outside hospital 3](#_Toc287594)

[Overview 3](#_Toc287595)

[Methods 3](#_Toc287596)

[Morbidity profile of patients with HNSCC discharged in 2008 4](#_Toc287597)

[Imputation of mortality outside hospital from 2008 to 2013 4](#_Toc287598)

[Cross-validation of mortality rates at 3 years 4](#_Toc287599)

[Reference List 4](#_Toc287600)

[Table 1. Morbidity profile of patients discharged with HNSCC in 2008, by vital status (N=40 811) 6](#_Toc287601)

[Table 2. Risk of in-hospital death in 2008 among patients with HNSCC and known vital status in 2008 (N=35 515) 9](#_Toc287602)

[Table 3. Vital status of patients discharged with HNSCC, by year 12](#_Toc287603)

[Table 4. Mortality rates at 3 years for HNSCC in the study population and French cancer registries 13](#_Toc287604)

[Additional Table 1. Coding dictionary 14](#_Toc287605)

[Reference List 15](#_Toc287606)

[Additional Table 2. Study flowchart 16](#_Toc287607)

[Additional Table 3. Eigenvalues of the Polychoric Correlation Matrix (two-parameter graded response model) 17](#_Toc287608)

[Additional Table 4. Parameter estimates (two-parameter graded response model) 18](#_Toc287609)

[Additional Table 5. Parameter estimates of the two-step selection model for “initial treatment at early stage” at 1 month of follow-up 19](#_Toc287610)

[Additional Table 6. Parameter estimates of the two-step selection model for “initial treatment at early stage” at 6 months of follow-up 21](#_Toc287611)

[Additional Table 7. Parameter estimates of the two-step selection model for “initial treatment at locally advanced stage” at 1 month of follow-up 23](#_Toc287612)

[Additional Table 8. Parameter estimates of the two-step selection model for “initial treatment at locally advanced stage” at 6 months of follow-up 25](#_Toc287613)

[Additional Table 9. Parameter estimates of the two-step selection model for “initial treatment with distant metastasis” at 1 month of follow-up 27](#_Toc287614)

[Additional Table 10. Parameter estimates of the two-step selection model for “initial treatment with distant metastasis” at 12 months of follow-up 29](#_Toc287615)

[Additional Table 11. Parameter estimates of the two-step selection model for “relapse treatment in the follow-up” at 1 month of follow-up 31](#_Toc287616)

[Additional Table 12. Parameter estimates of the two-step selection model for “relapse treatment in the follow-up” at 12 months of follow-up 33](#_Toc287617)

[Additional Table 13. Parameter estimates of the two-step selection model for “relapse-free in the follow-up” at 1 month of follow-up 35](#_Toc287618)

[Additional Table 14. Parameter estimates of the two-step selection model for “relapse-free in the follow-up” at 12 months of follow-up 37](#_Toc287619)

[Additional Table 15. Selection bias in post-acute care by health state and month of follow-up 39](#_Toc287620)

## Additional Methods. Imputation of mortality outside hospital

### Overview

Hospital admission remains frequent at end-of-life in France, overall and for cancer patients in particular. A majority of deaths occur at hospital, with stable figures over the last three decades [[1](#_ENREF_1_1)]. In addition, about two-third patients receive hospital care in the month preceding death [[2](#_ENREF_1_2), [3](#_ENREF_1_3)]. In cancer patients, the role of hospital is even exacerbated at end-of-life: death was recorded at hospital for 73% deceased patients in 1993-2008, without changes over time [[4](#_ENREF_1_4)], and above 90% patients received hospital care in the month preceding death in 2009 [[2](#_ENREF_1_2), [3](#_ENREF_1_3)]. We relied on this particular feature of the French healthcare system to impute mortality outside hospital among patients lost to follow-up based on their morbidity profile at last hospital discharge [[5](#_ENREF_1_5)].

### Methods

The French National Hospital Discharge database contains all public and private hospital claims for acute and post-acute care from 2008 to 2013. Accordingly, the vital status of each patient diagnosed with head and neck squamous-cell carcinoma (HNSCC) can be followed with use all his/her admissions to hospital until in-hospital death or last hospital discharge in 2008-2013. In patients alive at last hospital discharge, all hospital records are informative on their morbidity profile and therefore immediate mortality risk outside hospital [[6](#_ENREF_1_6)].

We used a sequential approach by year of hospital admission to impute mortality outside hospital among all patients with a diagnosis of HNSCC and lost to follow-up in the same year. Expectedly, patients admitted to hospital in 2008 had the lowest level of attrition as they may be followed at hospital over the next five years (2009-2013). Therefore, we characterized the morbidity profile of patients with known vital status in 2008 with use of all hospital records of 2008 and estimated the probability of death in a given year with use of multivariate logistic regression. Then we imputed mortality outside hospital at an optimal cut-off (min $\left\{ \sqrt{{(1-specificity)}^{2}+{(1-sensitivity)}^{2}} \right\}$) based on the morbidity profile of patients lost to follow-up at hospital from 2008 to 2013. Finally, because the proportion of patients lost to follow-up at hospital increases exponentially in 2013, we censored all information on vital status at July 1, 2013 in the study population selected in 2010-12, i.e., after the initial treatment period of six months necessary to characterize patients first diagnosed with HNSCC in December 2012.

To characterize morbidity profiles, a large amount of information was retrieved from all hospital records in a given year:

1. *Demographics*: gender, age in January, zip code of residency categorized in French regions.
2. *Primary cancer other than HNSCC*: lung cancer, esophageal cancer, and any other primary cancer [[7](#_ENREF_1_7)].
3. *Cancer stage*: distant metastasis (ICD-10 C78-C79), and otherwise lymph nodes (ICD-10 C77).
4. *Causes-of-death other than cancer.* We relied on the Global Burden of Disease study methodology to disentangle ICD-10 codes acceptable as an underlying cause-of-death from “garbage codes” [[8](#_ENREF_1_8), [9](#_ENREF_1_9)]. Acceptable causes-of-death and conditions that are intermediate causes of death (e.g., ICD-10 N17 for acute renal failure) were recorded by ICD-10 chapter. In absence of an acceptable cause-of-death, conditions that cannot be considered as the underlying cause-of-death were recorded by ICD-10 chapter (e.g., ICD-10 K02 codes for dental caries).
5. *Other prognostic factors*. Several factors of ICD-10 chapters XVIII and XXI were recorded: palliative care (ICD-10 Z51.5); chronic dialysis (medical procedures); transplantation status (ICD-10 Z94; T86 and medical procedures); poor general condition (ICD-10 R53.+0); shock (ICD-10 R57); bedridden (ICD-10 R26..30 or Z74.00); senility (ICD-10 R54).
6. *Hospital trajectory in the year*: total length of stay in acute inpatient care (in nights); admission in acute day case care (without inpatient care admission); emergency room admission; rehabilitation care admission; home care services provided by hospital; and nursing home admission.

In preliminary analyses, we found that patients lost to follow-up in palliative care or chronic dialysis had dramatic immediate mortality risks. Of 30,238 patients recorded in palliative care before October 2013, in-hospital death rate was 90% within 3 months. Of 618 patients on chronic dialysis at last discharge before October 2013, in-hospital death rate was 73%. We assumed that patients lost to follow-up in palliative care or chronic dialysis before October 2013 all died after hospital discharge.

### Morbidity profile of patients with HNSCC discharged in 2008

Of 40,811 patients discharged with HNSCC in 2008, 8,750 (21.4%) died at hospital in 2008, 26,765 (65.6%) were alive by the end of 2008 (i.e., they had hospital records in 2009-2013), and 5,296 (13.0%) were lost to follow-up at hospital in 2008 (**Table 1**). Patients lost to follow-up in 2008 were rather in better health than patients alive by the end of 2008 and even more so when compared to patients who died in 2008.

### Imputation of mortality outside hospital from 2008 to 2013

**Table 2** shows the results of the multivariate logistic regression on the probability of in-hospital death among 35,515 patients with known vital status in 2008. Overall, the concordance reached 88.5% between observed and predicted deaths in 2008. At the optimal cut-off (i.e., 20.5% predicted probability of death), sensitivity and specificity were 79.8% and 76.9%, respectively.

Mortality outside hospital was imputed at the optimal cut-off based on the morbidity profile of patients lost to follow-up at hospital from 2008 to 2013 (**Table 3**). Among 5,296 patients lost to follow-up at hospital in 2008, 1,450 (27.4%) deaths were imputed in 2008. While the proportion of patients lost to follow-up at hospital increased slightly from 2008 to 2012, it increased exponentially in 2013 and all information on vital status was censored at July 1, 2013.

### Cross-validation of mortality rates at 3 years

**Table 4** shows the mortality rates at 3 years estimated in the study population (Kaplan-Meier method) and reported in French cancer registries.[[10](#_ENREF_1_10)] Overall, mortality rates at 3 years estimated in the study population significantly increased from 43.6% (95% CI, 42.9-44.3) with use of in-hospital death records to 53.1% (95% CI 52.5-53.6) after imputation of mortality outside hospital and censoring at July 1, 2013. After imputation of mortality outside hospital, mortality rates at 3 years were similar between the study population and pooled estimates from French cancer registries, overall and by primary HNSCC site.

### Reference List

1. **Répartition des décès selon le lieu du décès [Distribution of deaths according to location]** [https://insee.fr/fr/statistiques/2117277?sommaire=2117290]

2. Pennec S, Monnier A, Pontone S, Aubry R: **End-of-life medical decisions in France: a death certificate follow-up survey 5 years after the 2005 act of parliament on patients' rights and end of life.** *BMC Palliat Care* 2012, **11:**25.

3. Observatoire National de la Fin de Vie: **Rapport 2012: vivre la fin de vie chez soi. [2012 Report: living end-of-life at home].** [**http://www.ladocumentationfrancaise.fr/rapports-publics/134000186-observatoire-national-de-la-fin-de-vie-rapport-2012-vivre-la-fin-de-sa-vie-chez-soi**](http://www.ladocumentationfrancaise.fr/rapports-publics/134000186-observatoire-national-de-la-fin-de-vie-rapport-2012-vivre-la-fin-de-sa-vie-chez-soi)**.** pp. 143: Observatoire National de la Fin de Vie,; 2013:143.

4. Gisquet E, Aouba A, Aubry R, Jougla E, Rey G: **Où meurt-on en France ? Analyse des certificats de décès (1993-2008) [Where do we die in France? Analysis of death certificates (1993-2008)].** [**http://invs.santepubliquefrance.fr/Publications-et-outils/BEH-Bulletin-epidemiologique-hebdomadaire/Archives/2012/BEH-n-48-2012**](http://invs.santepubliquefrance.fr/Publications-et-outils/BEH-Bulletin-epidemiologique-hebdomadaire/Archives/2012/BEH-n-48-2012)**.** *Bulletin Epidémiologique Hebdomadaire* 2012, **48:**547-551.

5. Schwarzinger M, Baillot S, Yazdanpanah Y, Rehm J, Mallet V: **Contribution of alcohol use disorders on the burden of chronic hepatitis C in France, 2008-2013: A nationwide retrospective cohort study.** *J Hepatol* 2017, **67:**454-461.

6. Schildcrout JS, Basford MA, Pulley JM, Masys DR, Roden DM, Wang D, Chute CG, Kullo IJ, Carrell D, Peissig P, et al: **An analytical approach to characterize morbidity profile dissimilarity between distinct cohorts using electronic medical records.** *J Biomed Inform* 2010, **43:**914-923.

7. Jegu J, Colonna M, Daubisse-Marliac L, Tretarre B, Ganry O, Guizard AV, Bara S, Troussard X, Bouvier V, Woronoff AS, Velten M: **The effect of patient characteristics on second primary cancer risk in France.** *BMC Cancer* 2014, **14:**94.

8. Lozano R, Naghavi M, Foreman K, Lim S, Shibuya K, Aboyans V, Abraham J, Adair T, Aggarwal R, Ahn SY, et al: **Global and regional mortality from 235 causes of death for 20 age groups in 1990 and 2010: a systematic analysis for the Global Burden of Disease Study 2010.** *Lancet* 2012, **380:**2095-2128.

9. Naghavi M, Makela S, Foreman K, O'Brien J, Pourmalek F, Lozano R: **Algorithms for enhancing public health utility of national causes-of-death data.** *Popul Health Metr* 2010, **8:**9.

10. Cowppli-Bony A, Uhry Z, Remontet L, Guizard A-V, Voirin N, Monnereau A, Bouvier A-M, Colonna M, Bossard N, Woronoff AS, Grosclaude P: **Survie des personnes atteintes de cancer en France métropolitaine, 1989-2013. Étude à partir des registres des cancers du réseau Francim. Partie 1 – Tumeurs solides.** Réseau français des registres des cancers (Francim), service de biostatistique des Hospices civils de Lyon (HCL), Institut de veille sanitaire (InVS), Institut national du cancer (INCa); 2016.

###

### Table 1. Morbidity profile of patients discharged with HNSCC in 2008, by vital status (N=40 811)

| **Morbidity profile** | | **In-hospital death in 2008** | **Alive by the end of 2008** | **Lost to follow-up in 2008** | **P-value** |
| --- | --- | --- | --- | --- | --- |
|  |  | **N=8 750** | **N=26 765** | **N=5 296** |  |
| **Men** | | 7 289 (83.3) | 21 725 (81.2) | 4 195 (79.2) | <0.0001 |
| **Age in January 2008, mean (std) years** | | 64.3 (11.9) | 61.4 (11.5) | 61.5 (12.8) | <0.0001 |
| **French region** | |  |  |  |  |
|  | Paris area | 1 337 (15.3) | 3 885 (14.5) | 818 (15.5) | <0.0001 |
|  | North-West | 2 268 (25.9) | 6 454 (24.1) | 1 516 (28.6) |  |
|  | North-East | 2 376 (27.2) | 7 117 (26.6) | 1 203 (22.7) |  |
|  | South-West | 819 (9.4) | 2 587 (9.7) | 467 (8.8) |  |
|  | South-East | 1 893 (21.6) | 6 555 (24.5) | 1 247 (23.6) |  |
|  | French West Indies | 57 (0.7) | 167 (0.6) | 57 (0.9) |  |
| **Other cancer (by ICD-10 chapter)** | |  |  |  |  |
|  | Lung cancer (II) | 1 072 (12.3) | 1 474 (5.5) | 207 (3.9) | <0.0001 |
|  | Esophageal cancer (II) | 531 (6.1) | 809 (3.0) | 115 (2.2) | <0.0001 |
|  | Cancer other than HNSCC lung or esophageal (II) | 1 688 (19.3) | 3 875 (14.5) | 606 (11.4) | <0.0001 |
|  | Metastasis (II) | 3 316 (37.9) | 2 345 (8.8) | 518 (9.8) | <0.0001 |
|  | Lymph nodes (without metastasis) (II) | 1 143 (13.1) | 4 314 (16.1) | 706 (13.3) | <0.0001 |
| **Other causes-of-death (by ICD-10 chapter)** | |  |  |  |  |
|  | HIV/AIDS (I) | 40 (0.5) | 115 (0.4) | 12 (0.2) | 0.078 |
|  | Infectious diseases other than HIV (I) | 2 506 (28.6) | 3 167 (11.8) | 573 (10.8) | <0.0001 |
|  | Blood disorders (III) | 1 964 (22.5) | 2 725 (10.2) | 482 (9.1) | <0.0001 |
|  | Malnutrition (IV) | 2 840 (32.5) | 3 915 (14.6) | 714 (13.5) | <0.0001 |
|  | Endocrine and metabolic disorders (IV) | 2 291 (26.2) | 6 073 (22.7) | 872 (16.5) | <0.0001 |
|  | Mental and behavioral disorders (V) | 2 578 (29.5) | 8 485 (31.7) | 1 326 (25.0) | <0.0001 |
|  | Diseases of the nervous system (VI) | 968 (11.1) | 1 589 (5.9) | 298 (5.6) | <0.0001 |
|  | Diseases of the circulatory system (IX) | 2 525 (28.9) | 6 183 (23.1) | 912 (17.2) | <0.0001 |
|  | Diseases of the respiratory system (X) | 2 086 (23.8) | 6 084 (22.7) | 936 (17.7) | <0.0001 |
|  | Diseases of the liver (XI) | 634 (7.3) | 1 257 (4.7) | 184 (3.5) | <0.0001 |
|  | Other diseases of the digestive system (XI) | 1 978 (22.6) | 5 316 (19.9) | 744 (14.1) | <0.0001 |
|  | Diseases of the skin and subcutaneous tissue (XII) | 551 (6.3) | 703 (2.6) | 125 (2.4) | <0.0001 |
|  | Diseases of the musculoskeletal system and connective tissue (XIII) | 333 (3.8) | 887 (3.3) | 111 (2.1) | <0.0001 |
|  | Diseases of the urinary system (XIV) | 473 (5.4) | 1 026 (3.8) | 129 (2.4) | <0.0001 |
|  | Gynecological diseases (XIV) | 6 (0.1) | 52 (0.2) | 10 (0.2) | <0.05 |
|  | External causes of mortality (XX) | 912 (10.4) | 1 963 (7.3) | 286 (5.4) | <0.0001 |
| **Intermediate causes-of-death (by ICD-10 chapter)** | |  |  |  |  |
|  | Infectious diseases other than HIV (I) | 607 (6.9) | 484 (1.8) | 91 (1.7) | <0.0001 |
|  | Endocrine and metabolic disorders (IV) | 841 (9.6) | 813 (3.0) | 159 (3.0) | <0.0001 |
|  | Diseases of the circulatory system (IX) | 555 (6.3) | 662 (2.5) | 92 (1.7) | <0.0001 |
|  | Diseases of the respiratory system (X) | 1 556 (17.8) | 1 140 (4.3) | 230 (4.3) | <0.0001 |
|  | Diseases of the liver (XI) | 183 (2.1) | 129 (0.5) | 19 (0.4) | <0.0001 |
|  | Diseases of the urinary system (XIV) | 804 (9.2) | 973 (3.6) | 165 (3.1) | <0.0001 |
| **Other prognostic factors (ICD-10 chapters XVIII and XXI)** | |  |  |  |  |
|  | Transplantation status | 31 (0.4) | 140 (0.5) | 11 (0.2) | <0.01 |
|  | Poor health condition | 2 568 (29.4) | 1 818 (6.8) | 428 (8.1) | <0.0001 |
|  | Shock | 2 022 (23.1) | 1 215 (4.5) | 208 (3.9) | <0.0001 |
|  | Bedridden | 515 (5.9) | 133 (0.5) | 52 (1.0) | <0.0001 |
|  | Senility | 48 (0.6) | 36 (0.1) | 7 (0.1) | <0.0001 |
| **Hospital trajectory (admission)** | |  |  |  |  |
|  | Inpatient care | 8 423 (96.3) | 23 694 (88.5) | 4 356 (82.3) | <0.0001 |
|  | Day case admission without inpatient care | 3 443 (39.4) | 13 013 (48.6) | 1 991 (37.6) | <0.0001 |
|  | Emergency room | 3 765 (43.0) | 5 027 (18.8) | 869 (16.4) | <0.0001 |
|  | Rehabilitation care | 2 033 (23.2) | 3 845 (14.4) | 553 (10.4) | <0.0001 |
|  | Home care services provided by hospital | 974 (11.1) | 784 (2.8) | 74 (1.4) | <0.0001 |
|  | Nursing home | 302 (3.5) | 361 (1.4) | 181 (3.4) | <0.0001 |

### Table 2. Risk of in-hospital death in 2008 among patients with HNSCC and known vital status in 2008 (N=35 515)

| **Morbidity profile** | | **Adjusted odds-ratio (95% CI)** | **P-value** |
| --- | --- | --- | --- |
| Men | | 1.15 (1.06-1.25) | <0.001 |
| Age | | 1.25 (1.13-1.38) | <0.0001 |
| Age^2^ | | 1.00 (0.99-1.00) | <0.0001 |
| Age^3^ | | 1.00 (1.00-1.00) | <0.0001 |
| French region (reference: Paris area) | |  |  |
|  | North-West | 1.28 (1.16-1.42) | <0.0001 |
|  | North-East | 1.12 (1.02-1.24) |  |
|  | South-West | 1.18 (1.04-1.33) |  |
|  | South-East | 1.11 (1.00-1.22) |  |
|  | French West Indies | 1,43 (1,01-2,03) |  |
| **Other cancer (by ICD-10 chapter)** | |  |  |
|  | Lung cancer (II) | 1.22 (1.10-1.36) | <0.001 |
|  | Esophageal cancer (II) | 1.42 (1.24-1.64) | <0.0001 |
|  | Cancer other than HNSCC, lung or esophageal (II) | 0.93 (0.86-1.01) | 0.073 |
|  | Metastasis (II) | 5.37 (4.98-5.79) | <0.0001 |
|  | Lymph nodes (without metastasis) (II) | 1.32 (1.21-1.43) | <0.0001 |
| **Other causes-of-death (by ICD-10 chapter)** | |  |  |
|  | HIV/AIDS (I) | 1.03 (0.65-1.63) | 0.90 |
|  | Infectious diseases other than HIV (I) | 1.46 (1.35-1.58) | <0.0001 |
|  | Blood disorders (III) | 1.31 (1.20-1.42) | <0.0001 |
|  | Malnutrition (IV) | 1.55 (1.44-1.66) | <0.0001 |
|  | Endocrine and metabolic disorders (IV) | 0.76 (0.71-0.82) | <0.0001 |
|  | Mental and behavioral disorders (V) | 0.72 (0.67-0.78) | <0.0001 |
|  | Diseases of the nervous system (VI) | 1.18 (1.06-1.32) | <0.01 |
|  | Diseases of the circulatory system (IX) | 0.90 (0.84-0.97) | <0.01 |
|  | Diseases of the respiratory system (X) | 0.97 (0.90-1.05) | 0.48 |
|  | Diseases of the liver (XI) | 1.64 (1.44-1.85) | <0.0001 |
|  | Other diseases of the digestive system (XI) | 0.72 (0.67-0.78) | <0.0001 |
|  | Diseases of the skin and subcutaneous tissue (XII) | 1.42 (1.23-1.65) | <0.0001 |
|  | Diseases of the musculoskeletal system and connective tissue (XIII) | 0.69 (0.58-0.81) | <0.0001 |
|  | Diseases of the urinary system (XIV) | 0.62 (0.54-0.72) | <0.0001 |
|  | Gynecological diseases (XIV) | 0.30 (0.11-0.81) | <0.05 |
|  | External causes of mortality (XX) | 0.77 (0.69-0.86) | <0.0001 |
| **Intermediate causes-of-death (by ICD-10 chapter)** | |  |  |
|  | Infectious diseases other than HIV (I) | 1.93 (1.65-2.25) | <0.0001 |
|  | Endocrine and metabolic disorders (IV) | 0.72 (0.63-0.83) | <0.0001 |
|  | Diseases of the circulatory system (IX) | 1.55 (1.34-1.80) | <0.0001 |
|  | Diseases of the respiratory system (X) | 2.83 (2.56-3.14) | <0.0001 |
|  | Diseases of the liver (XI) | 2.57 (1.95-3.40) | <0.0001 |
|  | Diseases of the urinary system (XIV) | 1.19 (1.05-1.35) | <0.01 |
| **Other prognostic factors (ICD-10 chapters XVIII and XXI)** | |  |  |
|  | Transplantation status | 0.76 (0.47-1.24) | 0.27 |
|  | Poor health condition | 3.20 (2.95-3.46) | <0.0001 |
|  | Shock | 3.96 (3.57-4.40) | <0.0001 |
|  | Bedridden | 4.77 (3.82-5.97) | <0.0001 |
|  | Senility | 1.87 (1.14-3.08) | <0.05 |
| **Hospital trajectory (admission)** | |  |  |
|  | Inpatient care | 1.39 (1.22-1.59) | <0.0001 |
|  | Day case admission without inpatient care | 0.54 (0.51-0.58) | <0.0001 |
|  | Emergency room | 2.15 (2.01-2.29) | <0.0001 |
|  | Rehabilitation care | 1.11 (1.03-1.20) | <0.05 |
|  | Home care services provided by hospital | 3.06 (2.71-3.45) | <0.0001 |
|  | Nursing home | 1.42 (1.17-1.72) | <0.001 |

### Table 3. Vital status of patients discharged with HNSCC, by year

| **Year** | **Total patients discharged in the year** | **New patients in the year** | **In-hospital death in the year** | **Alive at the end of the year** | **Patients lost to follow-up in the year** | | | **Increase in total deaths after imputation** |
| --- | --- | --- | --- | --- | --- | --- | --- | --- |
|  |  |  |  |  | **Total** | **Imputation of death status** | **Imputation of alive status** |  |
| 2008 | 40 811 | 40 811 (100) | 8 750 (21.4) | 26 765 (65.6) | 5 296 (13.0) | 1 450 (27.4) | 3 846 (72.6) | 1.17 |
| 2009 | 47 008 | 26 232 (55.8) | 9 693 (20.6) | 30 747 (65.4) | 6 568 (14.0) | 1 692 (25.8) | 4 876 (74.2) | 1.17 |
| 2010 | 50 224 | 23 158 (46.1) | 10 142 (20.2) | 32 345 (64.4) | 7 737 (15.4) | 1 793 (23.2) | 5 944 (76.8) | 1.18 |
| 2011 | 52 964 | 21 647 (40.9) | 10 372 (19.5) | 21 195 (60.8) | 10 397 (19.6) | 2 146 (20.6) | 8 251 (79.4) | 1.21 |
| 2012 | 55 074 | 20 505 (37.2) | 10 396 (18.8) | 28 521 (51.8) | 16 157 (29.3) | 2 922 (18.1) | 13 235 (81.1) | 1.28 |
| 2013 | 56 938 | 19 598 (34.4) | 10 383 (18.2) | 404 (0.7) | 46 151 (81.1) | 10 732 (23.3) | 35 419 (76.7) | 2.03 |

### Table 4. Mortality rates at 3 years for HNSCC in the study population and French cancer registries

| **Primary head and neck cancer site** | **Study population** | | | | |  | **French cancer registries (FRANCIM) 2005-2010** | | |
| --- | --- | --- | --- | --- | --- | --- | --- | --- | --- |
|  | **N** | **(%)** | **In-hospital death and follow-up to last discharge in 2010-2013** |  | **Overall death and complete follow-up to 07/01/2013** |  | **N** | **(%)** | **Survival** |
| Nasal cavity/paranasal sinuses (C30.0;C31) | 2 854 | 5.4% | 46.1 (42.5-49.6) |  | 56.4 (53.8-58.8) |  | 651 | 4.1% | 53 (49-57) |
| Nasopharynx (C11) | 1 781 | 3.3% | 52.9 (48.5-57.1) |  | 57.7 (54.9-60.5) |  | 283 | 1.8% | 61 (56-67) |
| Lip (C00) | 2 157 | 4.1% | 67.8 (63.0-72.2) |  | 73.1 (70.4-75.6) |  | 412 | 2.6% | 80 (76-84) |
| Tongue (C01-C02) | 7 826 | 14.7% | 40.4 (38.6-42.3) |  | 50.8 (49.4 (52.2) |  | 2 376 | 15.0% | 48 (46-50) |
| Oral cavity (C03-C06) | 8 215 | 15.4% | 43.8 (41.9-45.6) |  | 53.5 (52.1-54.9) |  | 2 847 | 18.0% | 53 (51-54) |
| Oropharynx (C09-C10) | 10 424 | 19.6% | 40.2 (38.5-41.9) |  | 50.5 (49.3-51.8) |  | 3 117 | 19.7% | 48 (46-50) |
| Hypopharynx (C12-C13) | 6 579 | 12.4% | 32.6 (30.7-34.5) |  | 40.9 (39.4-42.5) |  | 2 392 | 15.1% | 36 (34-38) |
| Ill-defined head and neck (C14) | 1 360 | 2.6% | 30.9 (27.1-34.8) |  | 36.4 (33.2-40.0) |  | 443 | 2.8% | -- |
| Head and neck (C01-C06; C09-C14) | 41 196 | 77.4% | 42.7 (41.9-43.4) |  | 52.0 (51.4-52.5) |  | 11 458 | 72.3% | 46 (45-47) |
| Larynx (C32) | 12 062 | 22.6% | 52.2 (50.6-53.8) |  | 59.7 (58.6-60.8) |  | 3 316 | 20.9% | 62 (60-64) |
| All head and neck | 53 258 | 100% | 43.6 (42.9-44.3) |  | 53.1 (52.5-53.6) |  | 15 837 | 100% | -- |

## Additional Table 1. Coding dictionary

| **Variables** | | | **International Classification of Diseases and Related Health Problems, 10th Revision, French version (ICD-10-FR)** | **References** |
| --- | --- | --- | --- | --- |
| **Patient selection** | | | | |
| Inclusion criteria: head and neck squamous-cell carcinoma (HNSCC) in 2008-2012 | | | C00-C06; C09-C14; C30.0; C31; C32 | [[1](#_ENREF_5_1)] |
| Exclusion criteria: | | |  |  |
|  | Any record of non-squamous cell carcinoma | |  |  |
|  |  | Kaposi sarcoma of the head and neck | C46.2; C46.70 |  |
|  |  | Middle ear | C30.1 |  |
|  |  | Salivary glands | C07-C08 |  |
|  |  | Skin cancer of lip or face | C44.0-C44.3 |  |
|  |  | Melanoma of lip or face | C43.0-C43.3 |  |
|  | Prevalent cases of HNSCC | | First diagnosis of HNSCC in 2008-2009 |  |
|  | Personal history of cancer | |  |  |
|  |  | Another cancer cared during the study period | Cxx recorded before first diagnosis of HNSCC |  |
|  |  | Another cancer cared before the study period | Z85 recorded at first diagnosis of HNSCC |  |
| **HNSCC characteristics at initial treatment (6 months after first diagnosis of HNSCC)** | | | | |
| Primary HNSCC site (primary discharge diagnosis) | | |  | [[1](#_ENREF_5_1)] |
|  | Nasopharynx | | C11 |  |
|  | Nasal cavity or paranasal sinuses | | C30.0; C31 |  |
|  | Lip | | C00 |  |
|  | Oral cavity (excluding base of tongue, soft palate, and uvula) | | C02-C04; C05.2; C05.8; C05.9; C06 |  |
|  | Oropharynx (including base of tongue, soft palate, and uvula) | | C01; C05.0; C05.1; C09-C10; C14.2 |  |
|  | Hypopharynx (including epilarynx) | | C12-C13; C32.1 |  |
|  | Larynx (excluding epilarynx) | | C32.0; C32.2; C32.3; C32.8; C32.9 |  |
|  | Ill-defined HNSCC | | C14.0; C14.8 |  |
| HNSCC stage | | |  | [[1](#_ENREF_5_1)] |
|  | Metastatic stage (any record of distant metastasis) | | C78-C79 |  |
|  | Locally advanced stage | |  |  |
|  |  | Any ICD-10 record indicating locoregional extension | C00.8; C02.8; C04.8; C05.8; C06.8; C08.8; C09.8; C10.8; C11.8; C13.8; C14.0; C14.8; C77 |  |
|  |  | Any treatment record eliminating an early stage | chemotherapy (Z51.1) surgery (HNSCC surgical procedures) and radiotherapy (Z51.0) tracheostomy at index HNSCC diagnosis (Z93.0, Z43.0, J95.0) palliative care (Z51.5) | [[2](#_ENREF_5_2), [3](#_ENREF_5_3)] |
|  | Early stage, by default | | -- |  |
| **Main risk factors of HNSCC** | | | | |
| Alcohol use disorders (AUD) | | |  | [[4](#_ENREF_5_4), [5](#_ENREF_5_5)] |
|  | Mental and behavioral disorders due to chronic harmful use of alcohol | | F10.1-F10.9; Z50.2 |  |
|  |  | Including alcohol dependency | F10.2-F10.9; Z50.2 |  |
|  |  | Including alcohol withdrawal or abstinence (see cautionary note**) | F10.20; F10.21; F10.22; F10.23; Z50.2 |  |
|  | Diseases due to harmful use of alcohol | | E24.4; E51.1; K70; G31.2; G62.1; G72.1; I42.6; K29.2; K85.2; K86.0 |  |
| Tobacco smoking | | | F17 | [[6](#_ENREF_5_6)] |
| **Comorbidities** | | | | |
| Second primary cancer | | |  | [[7](#_ENREF_5_7), [8](#_ENREF_5_8)] |
|  | HNSCC | | Any record at a different primary HNSCC site |  |
|  | Lung cancer | | C33-C34 |  |
|  | Esophageal cancer | | C15 |  |
|  | Other cancer | | Cxx other than HNSCC, lung cancer, esophagus cancer, or metastasis |  |
| Charlson comorbidity index other than cancer | | | ICD-10 coding algorithm validated for each comorbidity | [[9-12](#_ENREF_5_9)] |
|  | Including Human Immunodeficiency Virus (HIV) infection with Acquired Immune Deficiency Syndrome (AIDS) | | Z21; B20-B24 |  |
| Depression including suicide attempt | | | F32-F33; X6, X7, X80-X84 | [[13](#_ENREF_5_13)] |
| Palliative care | | | Z51.5 |  |

### Reference List

1. Amin MB, Edge S, Greene F, Byrd DR, Brookland RK, Washington MK, Gershenwald JE, Compton CC, Hess KR, Sullivan DC, et al: *AJCC Cancer Staging Manual, 8th ed.* New-York: Springer-Verlag; 2017.

2. Gregoire V, Lefebvre JL, Licitra L, Felip E, Group E-E-EGW: **Squamous cell carcinoma of the head and neck: EHNS-ESMO-ESTRO Clinical Practice Guidelines for diagnosis, treatment and follow-up.** *Ann Oncol* 2010, **21 Suppl 5:**v184-186.

3. Institut National du Cancer (INCa): **Algorithme de sélection des hospitalisations liées à la prise en charge du cancer dans les bases nationales d'activité hospitalière de court séjour « algorithme cancer » [Algorithm to select cancer-related hospitalizations in the French National Hospital Discharge (PMSI) database].** Boulogne-Billancourt: INCa; 2013.

4. Rehm J, Mathers C, Popova S, Thavorncharoensap M, Teerawattananon Y, Patra J: **Global burden of disease and injury and economic cost attributable to alcohol use and alcohol-use disorders.** *Lancet* 2009, **373:**2223-2233.

5. Schwarzinger M, Thiebaut SP, Baillot S, Mallet V, Rehm J: **Alcohol use disorders and associated chronic disease - a national retrospective cohort study from France.** *BMC Public Health* 2017, **18:**43.

6. GBD 2015 Risk Factors Collaborators: **Global, regional, and national comparative risk assessment of 79 behavioural, environmental and occupational, and metabolic risks or clusters of risks, 1990-2015: a systematic analysis for the Global Burden of Disease Study 2015.** *Lancet* 2016, **388:**1659-1724.

7. Working Group R: **International rules for multiple primary cancers (ICD-0 third edition).** *Eur J Cancer Prev* 2005, **14:**307-308.

8. Jegu J, Colonna M, Daubisse-Marliac L, Tretarre B, Ganry O, Guizard AV, Bara S, Troussard X, Bouvier V, Woronoff AS, Velten M: **The effect of patient characteristics on second primary cancer risk in France.** *BMC Cancer* 2014, **14:**94.

9. Charlson ME, Pompei P, Ales KL, MacKenzie CR: **A new method of classifying prognostic comorbidity in longitudinal studies: development and validation.** *J Chronic Dis* 1987, **40:**373-383.

10. Quan H, Sundararajan V, Halfon P, Fong A, Burnand B, Luthi JC, Saunders LD, Beck CA, Feasby TE, Ghali WA: **Coding algorithms for defining comorbidities in ICD-9-CM and ICD-10 administrative data.** *Med Care* 2005, **43:**1130-1139.

11. Quan H, Li B, Couris CM, Fushimi K, Graham P, Hider P, Januel JM, Sundararajan V: **Updating and validating the Charlson comorbidity index and score for risk adjustment in hospital discharge abstracts using data from 6 countries.** *Am J Epidemiol* 2011, **173:**676-682.

12. Bannay A, Chaignot C, Blotiere PO, Basson M, Weill A, Ricordeau P, Alla F: **The Best Use of the Charlson Comorbidity Index With Electronic Health Care Database to Predict Mortality.** *Med Care* 2016, **54:**188-194.

13. GBD 2015 Mortality and Causes of Death Collaborators: **Global, regional, and national life expectancy, all-cause mortality, and cause-specific mortality for 249 causes of death, 1980-2015: a systematic analysis for the Global Burden of Disease Study 2015.** *Lancet* 2016, **388:**1459-1544.

## Additional Table 2. Study flowchart

| **All adults residing in metropolitan France and discharged in 2008-2012 (French National Hospital Discharge database)** | | 27,284,709 |
| --- | --- | --- |
| **Selection: head and neck cancer (ICD-10: C00-C14; C30-C32)** | | 134,324 (0.49) |
| **Exclusion: any patient recorded with non-squamous cell carcinoma** | | 2,359 (1.76) |
|  | Salivary glands (ICD-10: C07-C08) | 1,427 (1.06) |
|  | Skin cancer of lip or face (ICD-10: C44.0-C44.3) | 708 (0.53) |
|  | Middle ear (ICD-10: C30.1) | 152 (0.11) |
|  | Kaposi sarcoma of the head and neck (ICD-10: C46.2; C46.70) | 52 (0.04) |
|  | Melanoma of lip or face (ICD-10: C43.0-C43.3) | 47 (0.03) |
| **Inclusion: HNSCC (ICD-10: C00-C06; C09-C14; C30.0; C31; C32) in 2008-2012** | | 131,965 (0.48) |
| **Exclusion: prevalent cases of HNSCC in 2008-2009 and cases with a personal history of cancer** | | 78,707 (59.64) |
|  | Exclusion of patients first diagnosed with HNSCC in 2008-2009 | 66,881 (50,68) |
|  | Exclusion of patients diagnosed with a cancer other than HNSCC before index diagnosis of HNSCC | 5,878 (4.45) |
|  | Exclusion of patients with a personal history of HNSCC (Z85) at first diagnosis of HNSCC in 2010-2012 | 2,918 (2.21) |
|  | Exclusion of patients with a personal history of cancer other than HNSCC (Z85) | 3,560 (2.70) |
| **Final study population: incident cases of HNSCC in 2010-2012** | | 53,258 (40.36) |

## Additional Table 3. Eigenvalues of the Polychoric Correlation Matrix (two-parameter graded response model)

|  | Eigenvalue | Difference | Proportion | Cumulative |
| --- | --- | --- | --- | --- |
| 1 | 4.00470433 | 3.13376145 | 0.6675 | 0.6675 |
| 2 | 0.87094288 | 0.22715728 | 0.1452 | 0.8126 |
| 3 | 0.64378560 | 0.45183166 | 0.1073 | 0.9199 |
| 4 | 0.19195395 | 0.02298036 | 0.0320 | 0.9519 |
| 5 | 0.16897359 | 0.04933393 | 0.0282 | 0.9801 |
| 6 | 0.11963965 |  | 0.0199 | 1.0000 |

## Additional Table 4. Parameter estimates (two-parameter graded response model)

| ADL | Parameter | Estimate | Standard Error | Pr > \|t\| |
| --- | --- | --- | --- | --- |
| 1. Dressing or bathing | Threshold 1 | -4.85786 | 0.03468 | <.001 |
|  | Threshold 2 | -2.10974 | 0.02187 | <.001 |
|  | Threshold 3 | 0.09221 | 0.01699 | <.001 |
|  | Slope | 5.50358 | 0.04189 | <.001 |
| 2. Functional mobility (transferring) | Threshold 1 | -3.57483 | 0.01922 | <.001 |
|  | Threshold 2 | -1.84661 | 0.01478 | <.001 |
|  | Threshold 3 | 0.07971 | 0.01268 | <.001 |
|  | Slope | 3.80551 | 0.02181 | <.001 |
| 3. Self-feeding | Threshold 1 | -1.05001 | 0.00702 | <.001 |
|  | Threshold 2 | -0.11530 | 0.00640 | <.001 |
|  | Threshold 3 | 1.03603 | 0.00702 | <.001 |
|  | Slope | 1.12121 | 0.00725 | <.001 |
| 4. Continence | Threshold 1 | -4.52007 | 0.02533 | <.001 |
|  | Threshold 2 | -3.03278 | 0.01986 | <.001 |
|  | Threshold 3 | -1.30038 | 0.01521 | <.001 |
|  | Slope | 4.10381 | 0.02582 | <.001 |
| 5. Social interaction | Threshold 1 | -3.50494 | 0.01437 | <.001 |
|  | Threshold 2 | -1.92953 | 0.00955 | <.001 |
|  | Threshold 3 | -0.02680 | 0.00740 | <.001 |
|  | Slope | 1.62220 | 0.00923 | <.001 |
| 6. Communication | Threshold 1 | -3.02945 | 0.01224 | <.001 |
|  | Threshold 2 | -1.58694 | 0.00843 | <.001 |
|  | Threshold 3 | 0.08805 | 0.00694 | <.001 |
|  | Slope | 1.40276 | 0.00830 | <.001 |

## Additional Table 5. Parameter estimates of the two-step selection model for “initial treatment at early stage” at 1 month of follow-up

| **Parameters** | **First step: probit regression of a patient recorded in post-acute care (n=15,750)** | | |  | **Second step: OLS regression of HSU in post-acute care (n=935)** | | |
| --- | --- | --- | --- | --- | --- | --- | --- |
|  | Estimate | Standard error | Pr > \|t\| |  | Estimate | Standard error | Pr > \|t\| |
| Intercept | -1.303780 | 0.098683 | <.0001 |  | 0.525473 | 0.096503 | <.0001 |
| Male | -0.248086 | 0.042969 | <.0001 |  | -0.015093 | 0.026913 | 0.5749 |
| Age at diagnosis: ≥80 | 0.645785 | 0.082995 | <.0001 |  | -0.329197 | 0.059727 | <.0001 |
| Age at diagnosis: 75-79 | 0.466304 | 0.088945 | <.0001 |  | -0.200469 | 0.061681 | 0.0012 |
| Age at diagnosis: 70-74 | 0.278190 | 0.092860 | 0.0027 |  | -0.234221 | 0.064059 | 0.0003 |
| Age at diagnosis: 65-69 | 0.277130 | 0.091457 | 0.0024 |  | -0.122165 | 0.063356 | 0.0538 |
| Age at diagnosis: 60-64 | 0.166689 | 0.088351 | 0.0592 |  | -0.110432 | 0.062356 | 0.0766 |
| Age at diagnosis: 55-59 | 0.031854 | 0.092474 | 0.7305 |  | -0.129396 | 0.065818 | 0.0493 |
| Age at diagnosis: 50-54 | 0.088141 | 0.095533 | 0.3562 |  | -0.007526 | 0.066671 | 0.9101 |
| Region of residence: North-West | 0.023718 | 0.060360 | 0.6944 |  | -- |  |  |
| Region of residence: North-East | -0.086459 | 0.061325 | 0.1586 |  | -- |  |  |
| Region of residence: South-West | 0.014568 | 0.075314 | 0.8466 |  | -- |  |  |
| Region of residence: South-East | 0.002253 | 0.058251 | 0.9692 |  | -- |  |  |
| Tobacco smoking | -0.126870 | 0.052820 | 0.0163 |  | -- |  |  |
| Heavy drinking | 0.269419 | 0.055472 | <.0001 |  | -- |  |  |
| Year at diagnosis: 2012 | -0.131893 | 0.045948 | 0.0041 |  | -- |  |  |
| Year at diagnosis: 2011 | -0.051313 | 0.043800 | 0.2414 |  | -- |  |  |
| Admission to public teaching hospital | -0.601033 | 0.043702 | <.0001 |  | -- |  |  |
| Admission to comprehensive cancer care center | -0.998298 | 0.115203 | <.0001 |  | -- |  |  |
| Admission to private clinic | -1.034163 | 0.058422 | <.0001 |  | -- |  |  |
| Nasal cavity/paranasal sinuses | -0.117509 | 0.072838 | 0.1067 |  | 0.002339 | 0.043024 | 0.9566 |
| Nasopharynx | 0.158821 | 0.102489 | 0.1212 |  | 0.057208 | 0.056869 | 0.3144 |
| Lip | -0.733784 | 0.088655 | <.0001 |  | -0.073472 | 0.064461 | 0.2544 |
| Oral cavity | -0.167913 | 0.054563 | 0.0021 |  | 0.013121 | 0.033555 | 0.6958 |
| Hypopharynx | -0.272236 | 0.076290 | 0.0004 |  | -0.051550 | 0.048575 | 0.2886 |
| Larynx | -0.155890 | 0.055303 | 0.0048 |  | -0.009094 | 0.033968 | 0.7889 |
| Synchronous secondary head and neck cancer | -0.319835 | 0.182439 | 0.0796 |  | 0.089315 | 0.132553 | 0.5004 |
| Cancer other than head and neck cancer | 0.381998 | 0.064493 | <.0001 |  | 0.054920 | 0.036681 | 0.1343 |
| Myocardial infarction | -0.055502 | 0.090761 | 0.5409 |  | -0.054447 | 0.054171 | 0.3149 |
| Congestive heart failure | 0.108281 | 0.058409 | 0.0638 |  | -0.021689 | 0.031797 | 0.4952 |
| Peripheral vascular disease | 0.054334 | 0.061896 | 0.3800 |  | -0.019046 | 0.034835 | 0.5845 |
| Cerebrovascular disease | 0.199952 | 0.062265 | 0.0013 |  | -0.078038 | 0.034581 | 0.0240 |
| Dementia | 0.517073 | 0.069705 | <.0001 |  | -0.165353 | 0.036654 | <.0001 |
| Chronic pulmonary disease | 0.073199 | 0.051280 | 0.1534 |  | -0.001784 | 0.029848 | 0.9523 |
| Rheumatologic disease | 0.398415 | 0.156985 | 0.0112 |  | -0.048699 | 0.076779 | 0.5259 |
| Peptic ulcer disease | 0.361007 | 0.108868 | 0.0009 |  | -0.043117 | 0.060623 | 0.4769 |
| Hemiplegia or paraplegia | 0.535174 | 0.094781 | <.0001 |  | -0.235679 | 0.049702 | <.0001 |
| Renal disease | 0.194035 | 0.078074 | 0.0129 |  | -0.015753 | 0.042161 | 0.7087 |
| Mild liver disease | 0.331607 | 0.084159 | <.0001 |  | 0.043662 | 0.050051 | 0.3830 |
| Moderate or severe liver disease | 0.036648 | 0.114950 | 0.7499 |  | -0.015604 | 0.067585 | 0.8174 |
| Diabetes without complication | -0.099225 | 0.067030 | 0.1388 |  | -0.013169 | 0.042713 | 0.7578 |
| Diabetes with complications | 0.237921 | 0.069514 | 0.0006 |  | -0.020563 | 0.038961 | 0.5976 |
| HIV/AIDS | 0.039302 | 0.260404 | 0.8800 |  | 0.180157 | 0.176692 | 0.3079 |
| Depression | 0.465244 | 0.059083 | <.0001 |  | -0.012442 | 0.033064 | 0.7067 |
| Lambda |  |  |  |  | 0.093089 | 0.038385 | 0.0153 |

Reference category: Female; 18-49 years old at diagnosis; residence in greater Paris area; primary oropharynx cancer in 2010; and no record of comorbidity.

## Additional Table 6. Parameter estimates of the two-step selection model for “initial treatment at early stage” at 6 months of follow-up

| **Parameters** | **First step: probit regression of a patient recorded in post-acute care (n=14,180)** | | |  | **Second step: OLS regression of HSU in post-acute care (n=255)** | | |
| --- | --- | --- | --- | --- | --- | --- | --- |
|  | Estimate | Standard error | Pr > \|t\| |  | Estimate | Standard error | Pr > \|t\| |
| Intercept | -2.273072 | 0.147183 | <.0001 |  | -0.452129 | 0.451144 | 0.3163 |
| Male | 0.002244 | 0.070023 | 0.9744 |  | 0.135181 | 0.053978 | 0.0123 |
| Age at diagnosis: ≥80 | 0.336029 | 0.125047 | 0.0072 |  | -0.061342 | 0.100137 | 0.5402 |
| Age at diagnosis: 75-79 | 0.349091 | 0.127291 | 0.0061 |  | -0.056507 | 0.103236 | 0.5841 |
| Age at diagnosis: 70-74 | 0.035159 | 0.140570 | 0.8025 |  | -0.332382 | 0.112506 | 0.0031 |
| Age at diagnosis: 65-69 | 0.086277 | 0.133661 | 0.5186 |  | -0.096333 | 0.103704 | 0.3529 |
| Age at diagnosis: 60-64 | 0.079983 | 0.123528 | 0.5173 |  | -0.101947 | 0.096165 | 0.2891 |
| Age at diagnosis: 55-59 | -0.142261 | 0.135418 | 0.2935 |  | -0.008951 | 0.111510 | 0.9360 |
| Age at diagnosis: 50-54 | 0.015318 | 0.132348 | 0.9079 |  | 0.079238 | 0.101047 | 0.4329 |
| Region of residence: North-West | -0.058440 | 0.090011 | 0.5162 |  | -- |  |  |
| Region of residence: North-East | -0.215716 | 0.093718 | 0.0213 |  | -- |  |  |
| Region of residence: South-West | -0.169065 | 0.114632 | 0.1403 |  | -- |  |  |
| Region of residence: South-East | -0.115692 | 0.086895 | 0.1831 |  | -- |  |  |
| Tobacco smoking | 0.016822 | 0.076832 | 0.8267 |  | -- |  |  |
| Heavy drinking | 0.261250 | 0.081635 | 0.0014 |  | -- |  |  |
| Year at diagnosis: 2012 | -0.055922 | 0.069270 | 0.4195 |  | -- |  |  |
| Year at diagnosis: 2011 | -0.118459 | 0.069084 | 0.0864 |  | -- |  |  |
| Admission to public teaching hospital | -0.041338 | 0.063818 | 0.5172 |  | -- |  |  |
| Admission to comprehensive cancer care center | -0.086040 | 0.103588 | 0.4062 |  | -- |  |  |
| Admission to private clinic | -0.145223 | 0.071089 | 0.0411 |  | -- |  |  |
| Nasal cavity/paranasal sinuses | -0.089265 | 0.110317 | 0.4184 |  | -0.176954 | 0.079913 | 0.0268 |
| Nasopharynx | 0.001646 | 0.165064 | 0.9920 |  | -0.182966 | 0.117014 | 0.1179 |
| Lip | -0.446751 | 0.129174 | 0.0005 |  | -0.178432 | 0.125651 | 0.1556 |
| Oral cavity | -0.172981 | 0.082904 | 0.0369 |  | -0.197385 | 0.067227 | 0.0033 |
| Hypopharynx | -0.195827 | 0.112793 | 0.0825 |  | -0.136531 | 0.093313 | 0.1434 |
| Larynx | -0.263831 | 0.087112 | 0.0025 |  | -0.171669 | 0.077067 | 0.0259 |
| Synchronous secondary head and neck cancer | 0.102095 | 0.204496 | 0.6176 |  | -0.078237 | 0.152719 | 0.6084 |
| Cancer other than head and neck cancer | 0.357123 | 0.087204 | <.0001 |  | 0.116863 | 0.082837 | 0.1583 |
| Myocardial infarction | 0.266689 | 0.108294 | 0.0138 |  | 0.168125 | 0.086549 | 0.0521 |
| Congestive heart failure | 0.157495 | 0.085839 | 0.0665 |  | -0.074503 | 0.069992 | 0.2871 |
| Peripheral vascular disease | 0.203116 | 0.081588 | 0.0128 |  | 0.039208 | 0.065169 | 0.5474 |
| Cerebrovascular disease | 0.218763 | 0.087728 | 0.0126 |  | 0.078481 | 0.068775 | 0.2538 |
| Dementia | 0.563072 | 0.104145 | <.0001 |  | -0.003429 | 0.113773 | 0.9760 |
| Chronic pulmonary disease | 0.123711 | 0.072785 | 0.0892 |  | 0.076035 | 0.057021 | 0.1824 |
| Rheumatologic disease | 0.034630 | 0.239913 | 0.8852 |  | 0.218232 | 0.164261 | 0.1840 |
| Peptic ulcer disease | 0.417550 | 0.139992 | 0.0029 |  | 0.022906 | 0.112729 | 0.8390 |
| Hemiplegia or paraplegia | 0.803257 | 0.117487 | <.0001 |  | -0.020251 | 0.128869 | 0.8751 |
| Renal disease | 0.102041 | 0.115560 | 0.3772 |  | -0.153446 | 0.082288 | 0.0622 |
| Mild liver disease | 0.117739 | 0.131435 | 0.3704 |  | 0.145058 | 0.094984 | 0.1267 |
| Moderate or severe liver disease | 0.494031 | 0.130915 | 0.0002 |  | 0.245742 | 0.117531 | 0.0365 |
| Diabetes without complication | -0.002743 | 0.098385 | 0.9778 |  | -0.106611 | 0.074531 | 0.1526 |
| Diabetes with complications | 0.139188 | 0.098123 | 0.1560 |  | -0.019277 | 0.068736 | 0.7791 |
| HIV/AIDS | -0.197346 | 0.426515 | 0.6436 |  | 0.266481 | 0.339964 | 0.4331 |
| Depression | 0.544331 | 0.078073 | <.0001 |  | 0.163016 | 0.094389 | 0.0842 |
| Lambda |  |  |  |  | 0.383672 | 0.165904 | 0.0207 |

Reference category: Female; 18-49 years old at diagnosis; residence in greater Paris area; primary oropharynx cancer in 2010; and no record of comorbidity.

## Additional Table 7. Parameter estimates of the two-step selection model for “initial treatment at locally advanced stage” at 1 month of follow-up

| **Parameters** | **First step: probit regression of a patient recorded in post-acute care (n=32,723)** | | |  | **Second step: OLS regression of HSU in post-acute care (n=1,851)** | | |
| --- | --- | --- | --- | --- | --- | --- | --- |
|  | Estimate | Standard error | Pr > \|t\| |  | Estimate | Standard error | Pr > \|t\| |
| Intercept | -1.791409 | 0.062341 | <.0001 |  | 0.423229 | 0.105057 | <.0001 |
| Male | -0.176258 | 0.030386 | <.0001 |  | -0.009501 | 0.020054 | 0.6357 |
| Age at diagnosis: ≥80 | 0.649225 | 0.056251 | <.0001 |  | -0.245048 | 0.041635 | <.0001 |
| Age at diagnosis: 75-79 | 0.374792 | 0.059828 | <.0001 |  | -0.178969 | 0.041113 | <.0001 |
| Age at diagnosis: 70-74 | 0.259545 | 0.058230 | <.0001 |  | -0.155470 | 0.040605 | 0.0001 |
| Age at diagnosis: 65-69 | 0.179837 | 0.055424 | 0.0012 |  | -0.096948 | 0.039347 | 0.0137 |
| Age at diagnosis: 60-64 | 0.151099 | 0.050242 | 0.0026 |  | -0.094332 | 0.036194 | 0.0092 |
| Age at diagnosis: 55-59 | 0.103602 | 0.049900 | 0.0379 |  | -0.027620 | 0.036056 | 0.4437 |
| Age at diagnosis: 50-54 | 0.125114 | 0.051453 | 0.0150 |  | -0.034017 | 0.037454 | 0.3638 |
| Region of residence: North-West | 0.007125 | 0.038427 | 0.8529 |  | -- |  |  |
| Region of residence: North-East | -0.163807 | 0.039419 | <.0001 |  | -- |  |  |
| Region of residence: South-West | -0.024595 | 0.050867 | 0.6287 |  | -- |  |  |
| Region of residence: South-East | -0.005040 | 0.038931 | 0.8970 |  | -- |  |  |
| Tobacco smoking | -0.001399 | 0.031175 | 0.9642 |  | -- |  |  |
| Heavy drinking | 0.169260 | 0.032469 | <.0001 |  | -- |  |  |
| Year at diagnosis: 2012 | 0.047348 | 0.030021 | 0.1148 |  | -- |  |  |
| Year at diagnosis: 2011 | 0.020727 | 0.030078 | 0.4908 |  | -- |  |  |
| Admission to public teaching hospital | -0.249618 | 0.029129 | <.0001 |  | -- |  |  |
| Admission to comprehensive cancer care center | -0.311138 | 0.046651 | <.0001 |  | -- |  |  |
| Admission to private clinic | -0.479754 | 0.031785 | <.0001 |  | -- |  |  |
| Nasal cavity/paranasal sinuses | 0.018358 | 0.065902 | 0.7806 |  | -0.066229 | 0.040928 | 0.1056 |
| Nasopharynx | 0.232713 | 0.066948 | 0.0005 |  | -0.015979 | 0.043821 | 0.7154 |
| Lip | 0.070148 | 0.124645 | 0.5736 |  | 0.099206 | 0.072851 | 0.1733 |
| Oral cavity | 0.045992 | 0.035710 | 0.1978 |  | 0.018922 | 0.023452 | 0.4197 |
| Hypopharynx | 0.065899 | 0.036133 | 0.0682 |  | -0.017044 | 0.024317 | 0.4834 |
| Larynx | 0.148548 | 0.038304 | 0.0001 |  | 0.000513 | 0.025372 | 0.9839 |
| Ill-defined HNSCC | 0.204299 | 0.059422 | 0.0006 |  | -0.074809 | 0.036837 | 0.0423 |
| Synchronous secondary head and neck cancer | 0.037915 | 0.047526 | 0.4250 |  | 0.044610 | 0.032209 | 0.1660 |
| Cancer other than head and neck cancer | 0.360800 | 0.036079 | <.0001 |  | 0.076093 | 0.024915 | 0.0023 |
| Myocardial infarction | -0.092640 | 0.068203 | 0.1744 |  | 0.063441 | 0.042001 | 0.1309 |
| Congestive heart failure | 0.078247 | 0.044682 | 0.0799 |  | -0.054886 | 0.025977 | 0.0346 |
| Peripheral vascular disease | 0.075462 | 0.041460 | 0.0687 |  | 0.028120 | 0.025732 | 0.2745 |
| Cerebrovascular disease | 0.121901 | 0.048939 | 0.0127 |  | -0.065991 | 0.029017 | 0.0230 |
| Dementia | 0.204117 | 0.066313 | 0.0021 |  | -0.172708 | 0.034074 | <.0001 |
| Chronic pulmonary disease | 0.167109 | 0.032157 | <.0001 |  | 0.012562 | 0.020452 | 0.5391 |
| Rheumatologic disease | 0.384564 | 0.131300 | 0.0034 |  | -0.007481 | 0.071223 | 0.9163 |
| Peptic ulcer disease | 0.139671 | 0.072891 | 0.0553 |  | 0.033947 | 0.042641 | 0.4260 |
| Hemiplegia or paraplegia | 0.328413 | 0.073375 | <.0001 |  | -0.211874 | 0.042781 | <.0001 |
| Renal disease | -0.027076 | 0.068418 | 0.6923 |  | -0.054853 | 0.038145 | 0.1504 |
| Mild liver disease | 0.089671 | 0.054805 | 0.1018 |  | -0.031851 | 0.034146 | 0.3509 |
| Moderate or severe liver disease | 0.057375 | 0.070683 | 0.4169 |  | 0.044237 | 0.044107 | 0.3159 |
| Diabetes without complication | -0.075965 | 0.044115 | 0.0851 |  | -0.002022 | 0.029867 | 0.9460 |
| Diabetes with complications | 0.171560 | 0.053048 | 0.0012 |  | 0.008928 | 0.031199 | 0.7748 |
| HIV/AIDS | 0.090099 | 0.164664 | 0.5843 |  | -0.140557 | 0.106133 | 0.1854 |
| Depression | 0.370244 | 0.041381 | <.0001 |  | 0.017714 | 0.027257 | 0.5158 |
| Palliative care | 0.766987 | 0.037188 | <.0001 |  | -0.308124 | 0.033585 | <.0001 |
| Lambda |  |  |  |  | 0.108291 | 0.043466 | 0.0127 |

Reference category: Female; 18-49 years old at diagnosis; residence in greater Paris area; primary oropharynx cancer in 2010; and no record of comorbidity.

## Additional Table 8. Parameter estimates of the two-step selection model for “initial treatment at locally advanced stage” at 6 months of follow-up

| **Parameters** | **First step: probit regression of a patient recorded in post-acute care (n=28,054)** | | |  | **Second step: OLS regression of HSU in post-acute care (n=2,108)** | | |
| --- | --- | --- | --- | --- | --- | --- | --- |
|  | Estimate | Standard error | Pr > \|t\| |  | Estimate | Standard error | Pr > \|t\| |
| Intercept | -1.854904 | 0.059478 | <.0001 |  | 0.238010 | 0.099777 | 0.0171 |
| Male | -0.109264 | 0.030918 | 0.0004 |  | 0.000053372 | 0.018504 | 0.9977 |
| Age at diagnosis: ≥80 | 0.484943 | 0.062331 | <.0001 |  | -0.167281 | 0.037219 | <.0001 |
| Age at diagnosis: 75-79 | 0.284222 | 0.061457 | <.0001 |  | -0.137250 | 0.037388 | 0.0002 |
| Age at diagnosis: 70-74 | 0.283888 | 0.055799 | <.0001 |  | -0.124222 | 0.034258 | 0.0003 |
| Age at diagnosis: 65-69 | 0.228272 | 0.050626 | <.0001 |  | -0.071634 | 0.031811 | 0.0243 |
| Age at diagnosis: 60-64 | 0.190278 | 0.045486 | <.0001 |  | -0.029615 | 0.029010 | 0.3073 |
| Age at diagnosis: 55-59 | 0.136087 | 0.044762 | 0.0024 |  | 0.002982 | 0.028645 | 0.9171 |
| Age at diagnosis: 50-54 | 0.109753 | 0.046407 | 0.0180 |  | 0.037172 | 0.029649 | 0.2099 |
| Region of residence: North-West | -0.224495 | 0.037178 | <.0001 |  | -- |  |  |
| Region of residence: North-East | -0.373060 | 0.037768 | <.0001 |  | -- |  |  |
| Region of residence: South-West | -0.308844 | 0.050047 | <.0001 |  | -- |  |  |
| Region of residence: South-East | -0.117891 | 0.036381 | 0.0012 |  | -- |  |  |
| Tobacco smoking | 0.104204 | 0.029336 | 0.0004 |  | -- |  |  |
| Heavy drinking | 0.224640 | 0.030272 | <.0001 |  | -- |  |  |
| Year at diagnosis: 2012 | -0.023795 | 0.029503 | 0.4199 |  | -- |  |  |
| Year at diagnosis: 2011 | 0.010315 | 0.028877 | 0.7209 |  | -- |  |  |
| Admission to public teaching hospital | 0.140827 | 0.025684 | <.0001 |  | -- |  |  |
| Admission to comprehensive cancer care center | 0.018181 | 0.028923 | 0.5296 |  | -- |  |  |
| Admission to private clinic | -0.064249 | 0.026514 | 0.0154 |  | -- |  |  |
| Nasal cavity/paranasal sinuses | -0.108325 | 0.071068 | 0.1274 |  | -0.069925 | 0.044864 | 0.1191 |
| Nasopharynx | 0.111000 | 0.068903 | 0.1072 |  | -0.067846 | 0.042847 | 0.1133 |
| Lip | -0.386248 | 0.175492 | 0.0277 |  | -0.155479 | 0.118070 | 0.1879 |
| Oral cavity | 0.021817 | 0.034457 | 0.5266 |  | -0.059732 | 0.021000 | 0.0044 |
| Hypopharynx | 0.145925 | 0.032857 | <.0001 |  | 0.001204 | 0.020197 | 0.9524 |
| Larynx | 0.072084 | 0.038089 | 0.0584 |  | 0.008823 | 0.023137 | 0.7029 |
| Ill-defined HNSCC | -0.121975 | 0.075787 | 0.1075 |  | -0.010304 | 0.047395 | 0.8279 |
| Synchronous secondary head and neck cancer | 0.177639 | 0.041236 | <.0001 |  | 0.020620 | 0.024679 | 0.4034 |
| Cancer other than head and neck cancer | 0.130400 | 0.032918 | <.0001 |  | 0.039895 | 0.019730 | 0.0432 |
| Myocardial infarction | -0.112412 | 0.062627 | 0.0727 |  | 0.001870 | 0.036829 | 0.9595 |
| Congestive heart failure | 0.100170 | 0.042683 | 0.0189 |  | -0.053468 | 0.024041 | 0.0261 |
| Peripheral vascular disease | 0.116355 | 0.036500 | 0.0014 |  | -0.010426 | 0.021194 | 0.6228 |
| Cerebrovascular disease | 0.045387 | 0.048986 | 0.3542 |  | -0.008511 | 0.026819 | 0.7510 |
| Dementia | 0.244612 | 0.079227 | 0.0020 |  | -0.177838 | 0.039138 | <.0001 |
| Chronic pulmonary disease | 0.242258 | 0.028646 | <.0001 |  | 0.047969 | 0.018695 | 0.0103 |
| Rheumatologic disease | 0.109653 | 0.145221 | 0.4502 |  | -0.059988 | 0.079797 | 0.4522 |
| Peptic ulcer disease | -0.021608 | 0.070195 | 0.7582 |  | 0.000284 | 0.039594 | 0.9943 |
| Hemiplegia or paraplegia | 0.556440 | 0.070194 | <.0001 |  | -0.189717 | 0.039149 | <.0001 |
| Renal disease | 0.067060 | 0.065335 | 0.3047 |  | -0.050801 | 0.036203 | 0.1606 |
| Mild liver disease | 0.094631 | 0.050471 | 0.0608 |  | -0.009564 | 0.028672 | 0.7387 |
| Moderate or severe liver disease | -0.060872 | 0.070947 | 0.3909 |  | -0.054538 | 0.040213 | 0.1750 |
| Diabetes without complication | 0.033296 | 0.042736 | 0.4359 |  | -0.003530 | 0.025805 | 0.8912 |
| Diabetes with complications | 0.130990 | 0.051297 | 0.0107 |  | -0.007982 | 0.028810 | 0.7817 |
| HIV/AIDS | 0.080120 | 0.156012 | 0.6076 |  | -0.019324 | 0.087677 | 0.8256 |
| Depression | 0.456738 | 0.036436 | <.0001 |  | 0.070066 | 0.025288 | 0.0056 |
| Palliative care | 0.934180 | 0.035960 | <.0001 |  | -0.133875 | 0.033856 | <.0001 |
| Lambda |  |  |  |  | 0.180892 | 0.043287 | <.0001 |

Reference category: Female; 18-49 years old at diagnosis; residence in greater Paris area; primary oropharynx cancer in 2010; and no record of comorbidity.

## Additional Table 9. Parameter estimates of the two-step selection model for “initial treatment with distant metastasis” at 1 month of follow-up

| **Parameters** | **First step: probit regression of a patient recorded in post-acute care (n=4,785)** | | |  | **Second step: OLS regression of HSU in post-acute care (n=452)** | | |
| --- | --- | --- | --- | --- | --- | --- | --- |
|  | Estimate | Standard error | Pr > \|t\| |  | Estimate | Standard error | Pr > \|t\| |
| Intercept | -1.531479 | 0.148369 | <.0001 |  | 0.155692 | 0.244873 | 0.5249 |
| Male | -0.132062 | 0.070371 | 0.0606 |  | -0.007732 | 0.047721 | 0.8713 |
| Age at diagnosis: ≥80 | 0.487707 | 0.134805 | 0.0003 |  | -0.187699 | 0.098962 | 0.0579 |
| Age at diagnosis: 75-79 | 0.412812 | 0.137505 | 0.0027 |  | -0.166489 | 0.098962 | 0.0925 |
| Age at diagnosis: 70-74 | 0.178928 | 0.138273 | 0.1957 |  | -0.215473 | 0.098625 | 0.0289 |
| Age at diagnosis: 65-69 | 0.136706 | 0.127784 | 0.2847 |  | -0.148222 | 0.092707 | 0.1099 |
| Age at diagnosis: 60-64 | 0.160567 | 0.119859 | 0.1804 |  | -0.070604 | 0.086490 | 0.4143 |
| Age at diagnosis: 55-59 | 0.127766 | 0.119061 | 0.2832 |  | -0.216023 | 0.086894 | 0.0129 |
| Age at diagnosis: 50-54 | 0.052235 | 0.125198 | 0.6765 |  | -0.066933 | 0.090480 | 0.4595 |
| Region of residence: North-West | -0.031032 | 0.082965 | 0.7084 |  | -- |  |  |
| Region of residence: North-East | -0.181762 | 0.084863 | 0.0322 |  | -- |  |  |
| Region of residence: South-West | -0.197501 | 0.115578 | 0.0875 |  | -- |  |  |
| Region of residence: South-East | 0.039845 | 0.085213 | 0.6401 |  | -- |  |  |
| Tobacco smoking | -0.016338 | 0.069138 | 0.8132 |  | -- |  |  |
| Heavy drinking | 0.148967 | 0.071544 | 0.0373 |  | -- |  |  |
| Year at diagnosis: 2012 | 0.039352 | 0.066925 | 0.5565 |  | -- |  |  |
| Year at diagnosis: 2011 | 0.095549 | 0.066034 | 0.1479 |  | -- |  |  |
| Admission to public teaching hospital | -0.265330 | 0.064728 | <.0001 |  | -- |  |  |
| Admission to comprehensive cancer care center | -0.358327 | 0.104540 | 0.0006 |  | -- |  |  |
| Admission to private clinic | -0.376524 | 0.072306 | <.0001 |  | -- |  |  |
| Nasal cavity/paranasal sinuses | -0.076318 | 0.126369 | 0.5459 |  | -0.061164 | 0.084356 | 0.4684 |
| Nasopharynx | 0.098312 | 0.139422 | 0.4807 |  | 0.010544 | 0.089963 | 0.9067 |
| Lip | 0.016860 | 0.261876 | 0.9487 |  | 0.098614 | 0.166809 | 0.5544 |
| Oral cavity | 0.035751 | 0.082121 | 0.6633 |  | 0.057380 | 0.054481 | 0.2922 |
| Hypopharynx | -0.082757 | 0.078188 | 0.2899 |  | -0.042855 | 0.055857 | 0.4429 |
| Larynx | -0.009265 | 0.085648 | 0.9139 |  | 0.006057 | 0.056389 | 0.9145 |
| Ill-defined HNSCC | 0.057674 | 0.133632 | 0.6660 |  | -0.203771 | 0.084941 | 0.0164 |
| Synchronous secondary head and neck cancer | -0.109465 | 0.113596 | 0.3352 |  | -0.028989 | 0.082666 | 0.7258 |
| Cancer other than head and neck cancer | 0.183343 | 0.059453 | 0.0020 |  | 0.094578 | 0.044388 | 0.0331 |
| Myocardial infarction | -0.270036 | 0.175671 | 0.1243 |  | -0.255374 | 0.122622 | 0.0373 |
| Congestive heart failure | 0.016254 | 0.100310 | 0.8713 |  | 0.092605 | 0.062881 | 0.1408 |
| Peripheral vascular disease | 0.064811 | 0.091145 | 0.4770 |  | 0.074194 | 0.057568 | 0.1975 |
| Cerebrovascular disease | 0.026249 | 0.109533 | 0.8106 |  | -0.019723 | 0.068184 | 0.7724 |
| Dementia | 0.304035 | 0.151796 | 0.0452 |  | -0.191212 | 0.091300 | 0.0362 |
| Chronic pulmonary disease | 0.150412 | 0.070239 | 0.0322 |  | -0.040065 | 0.048336 | 0.4072 |
| Rheumatologic disease | 0.134526 | 0.314760 | 0.6691 |  | 0.037492 | 0.196901 | 0.8490 |
| Peptic ulcer disease | 0.071384 | 0.169216 | 0.6731 |  | -0.078817 | 0.104212 | 0.4495 |
| Hemiplegia or paraplegia | 0.638766 | 0.125695 | <.0001 |  | -0.075316 | 0.088866 | 0.3967 |
| Renal disease | -0.293636 | 0.167612 | 0.0798 |  | -0.090474 | 0.109455 | 0.4085 |
| Mild liver disease | 0.039253 | 0.124896 | 0.7533 |  | 0.039843 | 0.082308 | 0.6283 |
| Moderate or severe liver disease | 0.045918 | 0.143705 | 0.7493 |  | -0.034008 | 0.091400 | 0.7098 |
| Diabetes without complication | 0.006325 | 0.096797 | 0.9479 |  | 0.079326 | 0.065032 | 0.2225 |
| Diabetes with complications | 0.082510 | 0.130783 | 0.5281 |  | -0.002629 | 0.079996 | 0.9738 |
| HIV/AIDS | 0.395778 | 0.405925 | 0.3296 |  | 0.116789 | 0.231594 | 0.6141 |
| Depression | 0.233286 | 0.094704 | 0.0138 |  | -0.063707 | 0.062421 | 0.3074 |
| Palliative care | 0.659951 | 0.063044 | <.0001 |  | -0.174356 | 0.068525 | 0.0109 |
| Lambda |  |  |  |  | 0.231389 | 0.110000 | 0.0354 |

Reference category: Female; 18-49 years old at diagnosis; residence in greater Paris area; primary oropharynx cancer in 2010; and no record of comorbidity.

## Additional Table 10. Parameter estimates of the two-step selection model for “initial treatment with distant metastasis” at 12 months of follow-up

| **Parameters** | **First step: probit regression of a patient recorded in post-acute care (n=1,387)** | | |  | **Second step: OLS regression of HSU in post-acute care (n=190)** | | |
| --- | --- | --- | --- | --- | --- | --- | --- |
|  | Estimate | Standard error | Pr > \|t\| |  | Estimate | Standard error | Pr > \|t\| |
| Intercept | -1.170855 | 0.244076 | <.0001 |  | 0.818752 | 0.282758 | 0.0038 |
| Male | 0.008825 | 0.129308 | 0.9456 |  | -0.017926 | 0.070662 | 0.7997 |
| Age at diagnosis: ≥80 | 0.367215 | 0.290102 | 0.2056 |  | -0.096037 | 0.150739 | 0.5241 |
| Age at diagnosis: 75-79 | 0.419449 | 0.252806 | 0.0971 |  | -0.204861 | 0.127568 | 0.1083 |
| Age at diagnosis: 70-74 | -0.064174 | 0.243909 | 0.7925 |  | -0.287340 | 0.130637 | 0.0278 |
| Age at diagnosis: 65-69 | 0.230270 | 0.197978 | 0.2448 |  | -0.189385 | 0.105616 | 0.0729 |
| Age at diagnosis: 60-64 | 0.025078 | 0.181033 | 0.8898 |  | -0.292902 | 0.097693 | 0.0027 |
| Age at diagnosis: 55-59 | 0.113758 | 0.177482 | 0.5216 |  | -0.245075 | 0.093400 | 0.0087 |
| Age at diagnosis: 50-54 | 0.109584 | 0.183751 | 0.5509 |  | -0.132325 | 0.097819 | 0.1761 |
| Region of residence: North-West | -0.312881 | 0.144430 | 0.0303 |  | -- |  |  |
| Region of residence: North-East | -0.437144 | 0.144424 | 0.0025 |  | -- |  |  |
| Region of residence: South-West | -0.428285 | 0.198860 | 0.0313 |  | -- |  |  |
| Region of residence: South-East | -0.318699 | 0.154395 | 0.0390 |  | -- |  |  |
| Tobacco smoking | 0.190661 | 0.115011 | 0.0974 |  | -- |  |  |
| Heavy drinking | 0.059096 | 0.116486 | 0.6119 |  | -- |  |  |
| Year at diagnosis: 2012 | -0.334112 | 0.142894 | 0.0194 |  | -- |  |  |
| Year at diagnosis: 2011 | -0.070400 | 0.104992 | 0.5025 |  | -- |  |  |
| Admission to public teaching hospital | -0.026684 | 0.104196 | 0.7979 |  | -- |  |  |
| Admission to comprehensive cancer care center | -0.044910 | 0.118256 | 0.7041 |  | -- |  |  |
| Admission to private clinic | -0.127513 | 0.106425 | 0.2309 |  | -- |  |  |
| Nasal cavity/paranasal sinuses | -0.308389 | 0.228573 | 0.1773 |  | -0.063833 | 0.126601 | 0.6141 |
| Nasopharynx | -0.626042 | 0.318197 | 0.0491 |  | -0.138747 | 0.179781 | 0.4403 |
| Lip | -0.925282 | 0.709147 | 0.1920 |  | -0.113280 | 0.364733 | 0.7561 |
| Oral cavity | -0.155985 | 0.142142 | 0.2725 |  | 0.040572 | 0.074016 | 0.5836 |
| Hypopharynx | -0.164577 | 0.125385 | 0.1893 |  | 0.069264 | 0.068633 | 0.3129 |
| Larynx | -0.296749 | 0.162433 | 0.0677 |  | 0.190309 | 0.094430 | 0.0439 |
| Ill-defined HNSCC | -0.216233 | 0.296486 | 0.4658 |  | 0.048695 | 0.161642 | 0.7632 |
| Synchronous secondary head and neck cancer | -0.161077 | 0.174411 | 0.3557 |  | 0.072503 | 0.096265 | 0.4514 |
| Cancer other than head and neck cancer | 0.079372 | 0.101103 | 0.4324 |  | -0.013099 | 0.054170 | 0.8089 |
| Myocardial infarction | 0.010441 | 0.248998 | 0.9666 |  | 0.122347 | 0.130555 | 0.3487 |
| Congestive heart failure | -0.034734 | 0.176224 | 0.8438 |  | -0.124665 | 0.092108 | 0.1759 |
| Peripheral vascular disease | 0.052560 | 0.146301 | 0.7194 |  | -0.005252 | 0.072308 | 0.9421 |
| Cerebrovascular disease | 0.138338 | 0.181707 | 0.4465 |  | 0.076064 | 0.094116 | 0.4190 |
| Dementia | 0.605619 | 0.349083 | 0.0828 |  | -0.240145 | 0.131712 | 0.0683 |
| Chronic pulmonary disease | 0.079453 | 0.112222 | 0.4789 |  | -0.029241 | 0.056515 | 0.6049 |
| Rheumatologic disease | 0.078856 | 0.732149 | 0.9142 |  | 0.889412 | 0.357550 | 0.0129 |
| Peptic ulcer disease | -0.221053 | 0.307764 | 0.4726 |  | -0.123952 | 0.169518 | 0.4647 |
| Hemiplegia or paraplegia | 0.422521 | 0.229464 | 0.0656 |  | -0.177408 | 0.099993 | 0.0760 |
| Renal disease | -0.099864 | 0.213357 | 0.6397 |  | 0.060617 | 0.116114 | 0.6016 |
| Mild liver disease | -0.453866 | 0.238119 | 0.0566 |  | -0.108466 | 0.135130 | 0.4222 |
| Moderate or severe liver disease | -0.638416 | 0.354537 | 0.0717 |  | -0.563594 | 0.250039 | 0.0242 |
| Diabetes without complication | 0.056178 | 0.179776 | 0.7547 |  | -0.040795 | 0.092091 | 0.6578 |
| Diabetes with complications | 0.197344 | 0.200326 | 0.3246 |  | -0.118839 | 0.097264 | 0.2218 |
| HIV/AIDS | -4.596202 | 378.666300 | 0.9903 |  | 0 |  |  |
| Depression | 0.498305 | 0.145968 | 0.0006 |  | -0.136104 | 0.091355 | 0.1363 |
| Palliative care | 1.081200 | 0.102293 | <.0001 |  | -0.297822 | 0.119899 | 0.0130 |
| Lambda |  |  |  |  | -0.038882 | 0.136954 | 0.7765 |

Reference category: Female; 18-49 years old at diagnosis; residence in greater Paris area; primary oropharynx cancer in 2010; and no record of comorbidity.

## Additional Table 11. Parameter estimates of the two-step selection model for “relapse treatment in the follow-up” at 1 month of follow-up

| **Parameters** | **First step: probit regression of a patient recorded in post-acute care (n=12,982)** | | |  | **Second step: OLS regression of HSU in post-acute care (n=1,515)** | | |
| --- | --- | --- | --- | --- | --- | --- | --- |
|  | Estimate | Standard error | Pr > \|t\| |  | Estimate | Standard error | Pr > \|t\| |
| Intercept | -1.683379 | 0.086584 | <.0001 |  | 0.098379 | 0.200779 | 0.6241 |
| Male | -0.141970 | 0.039786 | 0.0004 |  | -0.050919 | 0.025007 | 0.0417 |
| Age at diagnosis: ≥80 | 0.296487 | 0.080024 | 0.0002 |  | -0.249445 | 0.049049 | <.0001 |
| Age at diagnosis: 75-79 | 0.268519 | 0.076757 | 0.0005 |  | -0.215535 | 0.047476 | <.0001 |
| Age at diagnosis: 70-74 | 0.210364 | 0.069892 | 0.0026 |  | -0.138581 | 0.043686 | 0.0015 |
| Age at diagnosis: 65-69 | 0.082425 | 0.065271 | 0.2067 |  | -0.133226 | 0.040347 | 0.0010 |
| Age at diagnosis: 60-64 | 0.146176 | 0.056370 | 0.0095 |  | -0.075127 | 0.036352 | 0.0388 |
| Age at diagnosis: 55-59 | 0.059080 | 0.055402 | 0.2862 |  | -0.048819 | 0.035016 | 0.1633 |
| Age at diagnosis: 50-54 | 0.092372 | 0.056786 | 0.1038 |  | -0.011395 | 0.036325 | 0.7537 |
| Region of residence: North-West | -0.178382 | 0.048011 | 0.0002 |  | -- |  |  |
| Region of residence: North-East | -0.160508 | 0.047023 | 0.0006 |  | -- |  |  |
| Region of residence: South-West | -0.129758 | 0.063104 | 0.0398 |  | -- |  |  |
| Region of residence: South-East | -0.106453 | 0.047507 | 0.0250 |  | -- |  |  |
| Tobacco smoking | 0.084827 | 0.037613 | 0.0241 |  | -- |  |  |
| Heavy drinking | 0.161646 | 0.038443 | <.0001 |  | -- |  |  |
| Year at diagnosis: 2012 | -0.013120 | 0.041004 | 0.7490 |  | -- |  |  |
| Year at diagnosis: 2011 | 0.007209 | 0.034011 | 0.8321 |  | -- |  |  |
| Admission to public teaching hospital | -0.064170 | 0.032329 | 0.0472 |  | -- |  |  |
| Admission to comprehensive cancer care center | 0.022190 | 0.036684 | 0.5452 |  | -- |  |  |
| Admission to private clinic | -0.055123 | 0.032668 | 0.0915 |  | -- |  |  |
| Nasal cavity/paranasal sinuses | -0.052355 | 0.085838 | 0.5419 |  | -0.062125 | 0.054407 | 0.2535 |
| Nasopharynx | 0.236462 | 0.087047 | 0.0066 |  | -0.028436 | 0.053492 | 0.5950 |
| Lip | 0.077990 | 0.156472 | 0.6182 |  | 0.048782 | 0.099625 | 0.6244 |
| Oral cavity | -0.040177 | 0.043681 | 0.3577 |  | -0.018069 | 0.027137 | 0.5055 |
| Hypopharynx | 0.087252 | 0.042381 | 0.0395 |  | 0.038741 | 0.026063 | 0.1372 |
| Larynx | 0.187532 | 0.047827 | <.0001 |  | 0.095478 | 0.030534 | 0.0018 |
| Ill-defined HNSCC | -0.116690 | 0.116948 | 0.3184 |  | -0.121894 | 0.072554 | 0.0929 |
| Synchronous secondary head and neck cancer | 0.145998 | 0.053529 | 0.0064 |  | -0.012784 | 0.033509 | 0.7028 |
| Metachronous secondary head and neck cancer | 0.148349 | 0.068956 | 0.0314 |  | 0.043722 | 0.041929 | 0.2971 |
| Locally advanced stage at initial treatment | 0.197744 | 0.046329 | <.0001 |  | 0.077904 | 0.032874 | 0.0178 |
| Cancer other than head and neck cancer | 0.040824 | 0.035245 | 0.2468 |  | 0.024293 | 0.021531 | 0.2592 |
| Myocardial infarction | -0.053261 | 0.074224 | 0.4730 |  | -0.032113 | 0.043724 | 0.4627 |
| Congestive heart failure | 0.157824 | 0.052655 | 0.0027 |  | -0.041490 | 0.032262 | 0.1984 |
| Peripheral vascular disease | 0.103295 | 0.044958 | 0.0216 |  | -0.007340 | 0.027813 | 0.7919 |
| Cerebrovascular disease | 0.018879 | 0.059086 | 0.7493 |  | -0.010425 | 0.034434 | 0.7621 |
| Dementia | 0.293627 | 0.103496 | 0.0046 |  | -0.116657 | 0.057076 | 0.0410 |
| Chronic pulmonary disease | 0.126131 | 0.035928 | 0.0004 |  | 0.029583 | 0.023934 | 0.2165 |
| Rheumatologic disease | 0.081686 | 0.169537 | 0.6299 |  | 0.124470 | 0.098737 | 0.2074 |
| Peptic ulcer disease | 0.051284 | 0.081938 | 0.5314 |  | 0.011097 | 0.047247 | 0.8143 |
| Hemiplegia or paraplegia | 0.300490 | 0.084535 | 0.0004 |  | -0.146059 | 0.051237 | 0.0044 |
| Renal disease | 0.049674 | 0.080543 | 0.5374 |  | -0.018718 | 0.046258 | 0.6857 |
| Mild liver disease | 0.038875 | 0.062853 | 0.5362 |  | -0.016650 | 0.036723 | 0.6503 |
| Moderate or severe liver disease | -0.088710 | 0.083618 | 0.2887 |  | -0.085653 | 0.048905 | 0.0799 |
| Diabetes without complication | 0.011801 | 0.053892 | 0.8267 |  | -0.080500 | 0.032950 | 0.0146 |
| Diabetes with complications | 0.046455 | 0.065880 | 0.4807 |  | 0.010189 | 0.038328 | 0.7904 |
| HIV/AIDS | 0.278884 | 0.190970 | 0.1442 |  | 0.136045 | 0.108824 | 0.2112 |
| Depression | 0.406032 | 0.045421 | <.0001 |  | 0.091525 | 0.039524 | 0.0206 |
| Palliative care | 0.714264 | 0.035354 | <.0001 |  | -0.155334 | 0.053617 | 0.0038 |
| Lambda |  |  |  |  | 0.279303 | 0.092475 | 0.0025 |

Reference category: Female; 18-49 years old at diagnosis; residence in greater Paris area; primary oropharynx cancer in 2010; and no record of comorbidity.

## Additional Table 12. Parameter estimates of the two-step selection model for “relapse treatment in the follow-up” at 12 months of follow-up

| **Parameters** | **First step: probit regression of a patient recorded in post-acute care (n=4,303)** | | |  | **Second step: OLS regression of HSU in post-acute care (n=368)** | | |
| --- | --- | --- | --- | --- | --- | --- | --- |
|  | Estimate | Standard error | Pr > \|t\| |  | Estimate | Standard error | Pr > \|t\| |
| Intercept | -2.065958 | 0.167691 | <.0001 |  | 0.882826 | 0.344177 | 0.0103 |
| Male | 0.009914 | 0.082805 | 0.9047 |  | -0.062301 | 0.050287 | 0.2154 |
| Age at diagnosis: ≥80 | 0.371722 | 0.174945 | 0.0336 |  | -0.344399 | 0.106400 | 0.0012 |
| Age at diagnosis: 75-79 | 0.089701 | 0.170192 | 0.5982 |  | -0.070819 | 0.101098 | 0.4836 |
| Age at diagnosis: 70-74 | 0.252080 | 0.134382 | 0.0607 |  | -0.048246 | 0.080658 | 0.5497 |
| Age at diagnosis: 65-69 | 0.121481 | 0.123058 | 0.3236 |  | -0.055982 | 0.074447 | 0.4521 |
| Age at diagnosis: 60-64 | 0.021840 | 0.108370 | 0.8403 |  | -0.036495 | 0.063893 | 0.5679 |
| Age at diagnosis: 55-59 | -0.003230 | 0.103812 | 0.9752 |  | 0.041507 | 0.062076 | 0.5037 |
| Age at diagnosis: 50-54 | -0.042345 | 0.104981 | 0.6867 |  | -0.002315 | 0.063004 | 0.9707 |
| Region of residence: North-West | 0.029103 | 0.094706 | 0.7586 |  | -- |  |  |
| Region of residence: North-East | -0.055167 | 0.093346 | 0.5545 |  | -- |  |  |
| Region of residence: South-West | -0.076867 | 0.129938 | 0.5541 |  | -- |  |  |
| Region of residence: South-East | -0.039056 | 0.095363 | 0.6821 |  | -- |  |  |
| Tobacco smoking | 0.073454 | 0.074912 | 0.3268 |  | -- |  |  |
| Heavy drinking | 0.208042 | 0.075359 | 0.0058 |  | -- |  |  |
| Year at diagnosis: 2012 | -0.232167 | 0.628806 | 0.7120 |  | -- |  |  |
| Year at diagnosis: 2011 | -0.183661 | 0.063777 | 0.0040 |  | -- |  |  |
| Admission to public teaching hospital | -0.001472 | 0.063412 | 0.9815 |  | -- |  |  |
| Admission to comprehensive cancer care center | 0.087955 | 0.070336 | 0.2111 |  | -- |  |  |
| Admission to private clinic | -0.002218 | 0.063578 | 0.9722 |  | -- |  |  |
| Nasal cavity/paranasal sinuses | 0.229891 | 0.144948 | 0.1127 |  | 0.034848 | 0.087318 | 0.6898 |
| Nasopharynx | -0.044748 | 0.189444 | 0.8133 |  | 0.210019 | 0.123785 | 0.0898 |
| Lip | 0.201723 | 0.275436 | 0.4639 |  | 0.072632 | 0.170162 | 0.6695 |
| Oral cavity | -0.154516 | 0.092269 | 0.0940 |  | 0.000599 | 0.060435 | 0.9921 |
| Hypopharynx | 0.083480 | 0.085942 | 0.3314 |  | 0.040264 | 0.050985 | 0.4297 |
| Larynx | 0.234640 | 0.088806 | 0.0082 |  | 0.031694 | 0.054727 | 0.5625 |
| Ill-defined HNSCC | 0.040345 | 0.238964 | 0.8659 |  | -0.093277 | 0.143172 | 0.5147 |
| Synchronous secondary head and neck cancer | 0.018792 | 0.114568 | 0.8697 |  | 0.013299 | 0.067852 | 0.8446 |
| Metachronous secondary head and neck cancer | 0.276678 | 0.118791 | 0.0199 |  | 0.018944 | 0.071704 | 0.7916 |
| Locally advanced stage at initial treatment | 0.092761 | 0.083479 | 0.2665 |  | 0.013276 | 0.050628 | 0.7932 |
| Cancer other than head and neck cancer | 0.061748 | 0.068119 | 0.3647 |  | 0.023729 | 0.040712 | 0.5600 |
| Myocardial infarction | 0.081866 | 0.136699 | 0.5493 |  | 0.082966 | 0.073198 | 0.2570 |
| Congestive heart failure | 0.020165 | 0.106465 | 0.8498 |  | -0.027999 | 0.057793 | 0.6281 |
| Peripheral vascular disease | 0.191321 | 0.083524 | 0.0220 |  | 0.050321 | 0.051428 | 0.3278 |
| Cerebrovascular disease | 0.132187 | 0.112924 | 0.2418 |  | -0.104502 | 0.062922 | 0.0967 |
| Dementia | -0.111191 | 0.260048 | 0.6690 |  | 0.002349 | 0.144336 | 0.9870 |
| Chronic pulmonary disease | 0.197578 | 0.067911 | 0.0036 |  | -0.030892 | 0.045288 | 0.4952 |
| Rheumatologic disease | 0.394961 | 0.316304 | 0.2118 |  | -0.338407 | 0.160183 | 0.0346 |
| Peptic ulcer disease | 0.226466 | 0.151244 | 0.1343 |  | -0.110115 | 0.092111 | 0.2319 |
| Hemiplegia or paraplegia | 0.585730 | 0.152728 | 0.0001 |  | -0.362958 | 0.092499 | <.0001 |
| Renal disease | -0.064284 | 0.161148 | 0.6900 |  | 0.153100 | 0.090840 | 0.0919 |
| Mild liver disease | 0.022521 | 0.129918 | 0.8624 |  | 0.046928 | 0.072540 | 0.5177 |
| Moderate or severe liver disease | -0.087065 | 0.177467 | 0.6237 |  | -0.075451 | 0.104991 | 0.4724 |
| Diabetes without complication | 0.016026 | 0.109334 | 0.8835 |  | 0.033236 | 0.063441 | 0.6004 |
| Diabetes with complications | 0.216137 | 0.119270 | 0.0700 |  | -0.078631 | 0.069631 | 0.2588 |
| HIV/AIDS | 0.472916 | 0.387826 | 0.2227 |  | -0.281558 | 0.204316 | 0.1682 |
| Depression | 0.349320 | 0.085050 | <.0001 |  | -0.085993 | 0.060437 | 0.1548 |
| Palliative care | 0.982379 | 0.069827 | <.0001 |  | -0.413689 | 0.115082 | 0.0003 |
| Lambda |  |  |  |  | -0.103304 | 0.146081 | 0.4795 |

Reference category: Female; 18-49 years old at diagnosis; residence in greater Paris area; primary oropharynx cancer in 2010; and no record of comorbidity.

## Additional Table 13. Parameter estimates of the two-step selection model for “relapse-free in the follow-up” at 1 month of follow-up

| **Parameters** | **First step: probit regression of a patient recorded in post-acute care (n=27,368)** | | |  | **Second step: OLS regression of HSU in post-acute care (n=1,016)** | | |
| --- | --- | --- | --- | --- | --- | --- | --- |
|  | Estimate | Standard error | Pr > \|t\| |  | Estimate | Standard error | Pr > \|t\| |
| Intercept | -2.585567 | 0.086292 | <.0001 |  | -0.017542 | 0.237295 | 0.9411 |
| Male | -0.083548 | 0.039731 | 0.0355 |  | 0.031854 | 0.027517 | 0.2470 |
| Age at diagnosis: ≥80 | 0.481023 | 0.075814 | <.0001 |  | -0.164013 | 0.056416 | 0.0036 |
| Age at diagnosis: 75-79 | 0.424198 | 0.075916 | <.0001 |  | -0.149520 | 0.056352 | 0.0080 |
| Age at diagnosis: 70-74 | 0.278099 | 0.076081 | 0.0003 |  | -0.159118 | 0.054351 | 0.0034 |
| Age at diagnosis: 65-69 | 0.270091 | 0.070128 | 0.0001 |  | -0.064795 | 0.051579 | 0.2090 |
| Age at diagnosis: 60-64 | 0.232936 | 0.064819 | 0.0003 |  | -0.052327 | 0.048752 | 0.2831 |
| Age at diagnosis: 55-59 | 0.107538 | 0.065673 | 0.1015 |  | -0.028727 | 0.048073 | 0.5501 |
| Age at diagnosis: 50-54 | 0.120594 | 0.067722 | 0.0750 |  | 0.061189 | 0.049662 | 0.2179 |
| Region of residence: North-West | -0.116957 | 0.050581 | 0.0208 |  | -- |  |  |
| Region of residence: North-East | -0.218458 | 0.050872 | <.0001 |  | -- |  |  |
| Region of residence: South-West | -0.295834 | 0.068311 | <.0001 |  | -- |  |  |
| Region of residence: South-East | -0.071075 | 0.049314 | 0.1495 |  | -- |  |  |
| Tobacco smoking | 0.103796 | 0.039490 | 0.0086 |  | -- |  |  |
| Heavy drinking | 0.196957 | 0.041551 | <.0001 |  | -- |  |  |
| Year at diagnosis: 2012 | -0.025412 | 0.038730 | 0.5117 |  | -- |  |  |
| Year at diagnosis: 2011 | -0.031425 | 0.039473 | 0.4260 |  | -- |  |  |
| Admission to public teaching hospital | 0.096380 | 0.034434 | 0.0051 |  | -- |  |  |
| Admission to comprehensive cancer care center | 0.064570 | 0.040397 | 0.1100 |  | -- |  |  |
| Admission to private clinic | -0.049131 | 0.036326 | 0.1762 |  | -- |  |  |
| Nasal cavity/paranasal sinuses | -0.064322 | 0.080611 | 0.4249 |  | -0.032283 | 0.056539 | 0.5680 |
| Nasopharynx | 0.094644 | 0.094235 | 0.3152 |  | -0.013525 | 0.065559 | 0.8366 |
| Lip | -0.126501 | 0.098401 | 0.1986 |  | -0.127234 | 0.076868 | 0.0979 |
| Oral cavity | 0.004884 | 0.046756 | 0.9168 |  | -0.059488 | 0.031705 | 0.0606 |
| Hypopharynx | 0.128866 | 0.047959 | 0.0072 |  | 0.028169 | 0.033590 | 0.4017 |
| Larynx | 0.060485 | 0.048678 | 0.2140 |  | 0.045982 | 0.033375 | 0.1683 |
| Ill-defined HNSCC | -0.165800 | 0.109626 | 0.1304 |  | -0.038325 | 0.075683 | 0.6126 |
| Synchronous secondary head and neck cancer | 0.214089 | 0.063192 | 0.0007 |  | 0.030290 | 0.043143 | 0.4826 |
| Metachronous secondary head and neck cancer | 0.393519 | 0.180231 | 0.0290 |  | 0.115130 | 0.112942 | 0.3080 |
| Locally advanced stage at initial treatment | 0.379640 | 0.040692 | <.0001 |  | 0.050102 | 0.040918 | 0.2208 |
| Cancer other than head and neck cancer | 0.213395 | 0.045875 | <.0001 |  | 0.050809 | 0.033000 | 0.1236 |
| Myocardial infarction | 0.039278 | 0.071151 | 0.5809 |  | 0.042648 | 0.044991 | 0.3432 |
| Congestive heart failure | 0.133589 | 0.051228 | 0.0091 |  | -0.044363 | 0.032511 | 0.1724 |
| Peripheral vascular disease | 0.175431 | 0.046522 | 0.0002 |  | 0.053216 | 0.032419 | 0.1007 |
| Cerebrovascular disease | 0.112451 | 0.056960 | 0.0484 |  | 0.056037 | 0.035581 | 0.1153 |
| Dementia | 0.367981 | 0.078538 | <.0001 |  | -0.167649 | 0.052216 | 0.0013 |
| Chronic pulmonary disease | 0.208845 | 0.038205 | <.0001 |  | 0.032277 | 0.029314 | 0.2709 |
| Rheumatologic disease | 0.074769 | 0.167350 | 0.6550 |  | -0.124935 | 0.099307 | 0.2084 |
| Peptic ulcer disease | -0.055778 | 0.095350 | 0.5586 |  | -0.053400 | 0.061512 | 0.3853 |
| Hemiplegia or paraplegia | 0.640100 | 0.079446 | <.0001 |  | -0.172767 | 0.060568 | 0.0043 |
| Renal disease | 0.151475 | 0.072140 | 0.0358 |  | 0.049824 | 0.045973 | 0.2785 |
| Mild liver disease | 0.167383 | 0.068827 | 0.0150 |  | 0.057240 | 0.046589 | 0.2192 |
| Moderate or severe liver disease | 0.011447 | 0.094097 | 0.9032 |  | 0.009988 | 0.057790 | 0.8628 |
| Diabetes without complication | 0.027428 | 0.056507 | 0.6274 |  | -0.033855 | 0.038490 | 0.3791 |
| Diabetes with complications | 0.121504 | 0.060903 | 0.0460 |  | 0.007918 | 0.037931 | 0.8346 |
| HIV/AIDS | 0.075703 | 0.204145 | 0.7108 |  | -0.048959 | 0.132355 | 0.7115 |
| Depression | 0.498525 | 0.045327 | <.0001 |  | 0.080569 | 0.043817 | 0.0659 |
| Palliative care | 1.012810 | 0.049793 | <.0001 |  | -0.128949 | 0.067493 | 0.0561 |
| Lambda |  |  |  |  | 0.219333 | 0.081957 | 0.0074 |

Reference category: Female; 18-49 years old at diagnosis; residence in greater Paris area; primary oropharynx cancer in 2010; and no record of comorbidity.

## Additional Table 14. Parameter estimates of the two-step selection model for “relapse-free in the follow-up” at 12 months of follow-up

| **Parameters** | **First step: probit regression of a patient recorded in post-acute care (n=16,511)** | | |  | **Second step: OLS regression of HSU in post-acute care (n=290)** | | |
| --- | --- | --- | --- | --- | --- | --- | --- |
|  | Estimate | Standard error | Pr > \|t\| |  | Estimate | Standard error | Pr > \|t\| |
| Intercept | -2.550633 | 0.136527 | <.0001 |  | 0.432345 | 0.510877 | 0.3974 |
| Male | -0.074913 | 0.067180 | 0.2648 |  | -0.049676 | 0.048434 | 0.3051 |
| Age at diagnosis: ≥80 | 0.405757 | 0.111322 | 0.0003 |  | -0.223020 | 0.085593 | 0.0092 |
| Age at diagnosis: 75-79 | 0.016962 | 0.124593 | 0.8917 |  | -0.216797 | 0.090829 | 0.0170 |
| Age at diagnosis: 70-74 | -0.045329 | 0.125284 | 0.7175 |  | -0.207302 | 0.094136 | 0.0277 |
| Age at diagnosis: 65-69 | -0.051961 | 0.115026 | 0.6515 |  | -0.172137 | 0.081665 | 0.0350 |
| Age at diagnosis: 60-64 | -0.208754 | 0.108414 | 0.0542 |  | -0.118583 | 0.088913 | 0.1823 |
| Age at diagnosis: 55-59 | -0.036082 | 0.099998 | 0.7182 |  | 0.007109 | 0.073093 | 0.9225 |
| Age at diagnosis: 50-54 | -0.178079 | 0.109407 | 0.1036 |  | 0.067568 | 0.080541 | 0.4015 |
| Region of residence: North-West | 0.038441 | 0.094356 | 0.6837 |  | -- |  |  |
| Region of residence: North-East | 0.056226 | 0.091228 | 0.5377 |  | -- |  |  |
| Region of residence: South-West | -0.028791 | 0.114624 | 0.8017 |  | -- |  |  |
| Region of residence: South-East | 0.118254 | 0.089576 | 0.1868 |  | -- |  |  |
| Tobacco smoking | 0.090034 | 0.068590 | 0.1893 |  | -- |  |  |
| Heavy drinking | 0.213653 | 0.073293 | 0.0036 |  | -- |  |  |
| Year at diagnosis: 2012 | -0.149156 | 0.283218 | 0.5984 |  | -- |  |  |
| Year at diagnosis: 2011 | -0.032164 | 0.054129 | 0.5524 |  | -- |  |  |
| Admission to public teaching hospital | 0.046625 | 0.059353 | 0.4321 |  | -- |  |  |
| Admission to comprehensive cancer care center | -0.060335 | 0.079165 | 0.4460 |  | -- |  |  |
| Admission to private clinic | -0.022009 | 0.061858 | 0.7220 |  | -- |  |  |
| Nasal cavity/paranasal sinuses | -0.047267 | 0.132836 | 0.7220 |  | 0.017418 | 0.089018 | 0.8449 |
| Nasopharynx | 0.039775 | 0.167267 | 0.8120 |  | -0.109266 | 0.114064 | 0.3381 |
| Lip | -0.137110 | 0.134259 | 0.3071 |  | 0.040325 | 0.094055 | 0.6681 |
| Oral cavity | -0.075346 | 0.083164 | 0.3649 |  | 0.036315 | 0.058757 | 0.5365 |
| Hypopharynx | 0.053932 | 0.091029 | 0.5535 |  | -0.004573 | 0.066043 | 0.9448 |
| Larynx | 0.123081 | 0.079473 | 0.1215 |  | 0.112546 | 0.057032 | 0.0485 |
| Ill-defined HNSCC | -0.105643 | 0.197367 | 0.5925 |  | 0.044207 | 0.137889 | 0.7485 |
| Synchronous secondary head and neck cancer | 0.100436 | 0.130096 | 0.4401 |  | -0.063504 | 0.092166 | 0.4908 |
| Metachronous secondary head and neck cancer | 0.709750 | 0.253199 | 0.0051 |  | -0.047376 | 0.181726 | 0.7943 |
| Locally advanced stage at initial treatment | -0.105997 | 0.061333 | 0.0839 |  | 0.054296 | 0.046388 | 0.2418 |
| Cancer other than head and neck cancer | 0.132057 | 0.078075 | 0.0908 |  | -0.151907 | 0.056402 | 0.0071 |
| Myocardial infarction | 0.007383 | 0.109351 | 0.9462 |  | 0.039064 | 0.068029 | 0.5658 |
| Congestive heart failure | 0.314911 | 0.078744 | <.0001 |  | -0.031466 | 0.070410 | 0.6550 |
| Peripheral vascular disease | 0.250523 | 0.073460 | 0.0006 |  | 0.057238 | 0.063324 | 0.3661 |
| Cerebrovascular disease | 0.038713 | 0.087904 | 0.6596 |  | -0.022447 | 0.052787 | 0.6707 |
| Dementia | 0.390165 | 0.115531 | 0.0007 |  | -0.338746 | 0.090125 | 0.0002 |
| Chronic pulmonary disease | 0.228107 | 0.062833 | 0.0003 |  | 0.107494 | 0.063420 | 0.0901 |
| Rheumatologic disease | 0.624917 | 0.186989 | 0.0008 |  | -0.070453 | 0.145010 | 0.6271 |
| Peptic ulcer disease | 0.072625 | 0.140301 | 0.6047 |  | -0.153912 | 0.089623 | 0.0859 |
| Hemiplegia or paraplegia | 0.843102 | 0.108255 | <.0001 |  | -0.153152 | 0.143472 | 0.2858 |
| Renal disease | 0.186843 | 0.111322 | 0.0933 |  | -0.158909 | 0.073512 | 0.0306 |
| Mild liver disease | 0.388687 | 0.103843 | 0.0002 |  | 0.109247 | 0.099067 | 0.2701 |
| Moderate or severe liver disease | 0.428141 | 0.124427 | 0.0006 |  | 0.162674 | 0.105650 | 0.1236 |
| Diabetes without complication | -0.138886 | 0.110881 | 0.2104 |  | -0.077313 | 0.086839 | 0.3733 |
| Diabetes with complications | 0.159107 | 0.091153 | 0.0809 |  | -0.039078 | 0.060880 | 0.5209 |
| HIV/AIDS | 0.163799 | 0.316423 | 0.6047 |  | 0.357992 | 0.228748 | 0.1176 |
| Depression | 0.508625 | 0.071418 | <.0001 |  | -0.034265 | 0.097528 | 0.7253 |
| Palliative care | 0.765169 | 0.111695 | <.0001 |  | -0.205303 | 0.133466 | 0.1240 |
| Lambda |  |  |  |  | 0.094981 | 0.187031 | 0.6116 |

Reference category: Female; 18-49 years old at diagnosis; residence in greater Paris area; primary oropharynx cancer in 2010; and no record of comorbidity.

## Additional Table 15. Selection bias in post-acute care by health state and month of follow-up

| **Month of follow-up** | **Initial treatment at early stage** | | | **Initial treatment at locally advanced stage** | | | **Initial treatment with distant metastasis** | | | **Relapse treatment in the follow-up** | | | **Relapse-free in the follow-up** | | |
| --- | --- | --- | --- | --- | --- | --- | --- | --- | --- | --- | --- | --- | --- | --- | --- |
|  | Est. | Std error | Pr > \|t\| | Est. | Std error | Pr > \|t\| | Est. | Std error | Pr > \|t\| | Est. | Std error | Pr > \|t\| | Est. | Std error | Pr > \|t\| |
| 1 | 0.09 | 0.04 | **<0.05** | 0.11 | 0.04 | **<0.05** | 0.23 | 0.11 | **<0.05** | 0.28 | 0.09 | **<0.01** | 0.22 | 0.08 | **<0.01** |
| 2 | 0.10 | 0.05 | **<0.05** | 0.19 | 0.04 | **<0.001** | 0.06 | 0.09 | 0.50 | 0.15 | 0.07 | **<0.05** | 0.22 | 0.09 | **<0.05** |
| 3 | 0.14 | 0.07 | **<0.05** | 0.19 | 0.03 | **<0.001** | 0.02 | 0.08 | 0.82 | 0.15 | 0.07 | **<0.05** | 0.20 | 0.11 | 0.06 |
| 4 | 0.14 | 0.09 | 0.14 | 0.17 | 0.03 | **<0.001** | -0.01 | 0.08 | 0.94 | 0.07 | 0.09 | 0.44 | 0.21 | 0.12 | 0.07 |
| 5 | 0.28 | 0.16 | 0.09 | 0.16 | 0.03 | **<0.001** | 0.03 | 0.08 | 0.73 | -0.01 | 0.10 | 0.90 | 0.19 | 0.14 | 0.16 |
| 6 | 0.38 | 0.17 | **<0.05** | 0.18 | 0.04 | **<0.001** | 0.01 | 0.08 | 0.92 | -0.05 | 0.09 | 0.57 | 0.28 | 0.16 | 0.08 |
| 7 |  |  |  |  |  |  | -0.03 | 0.09 | 0.76 | -0.09 | 0.10 | 0.35 | -0.27 | 0.15 | 0.08 |
| 8 |  |  |  |  |  |  | -0.09 | 0.10 | 0.39 | -0.08 | 0.10 | 0.44 | -0.01 | 0.17 | 0.95 |
| 9 |  |  |  |  |  |  | -0.07 | 0.11 | 0.49 | -0.29 | 0.14 | **<0.05** | 0.17 | 0.17 | 0.31 |
| 10 |  |  |  |  |  |  | 0.01 | 0.11 | 0.93 | -0.08 | 0.13 | 0.52 | 0.09 | 0.16 | 0.57 |
| 11 |  |  |  |  |  |  | -0.01 | 0.13 | 0.94 | 0.04 | 0.14 | 0.75 | 0.12 | 0.17 | 0.48 |
| 12 |  |  |  |  |  |  | -0.04 | 0.14 | 0.78 | -0.10 | 0.15 | 0.48 | 0.09 | 0.19 | 0.61 |
